# Structural Receptors and Host Adaptations Affecting the Bacteriophage Targeting of *Vibrio* Pathogens

**DOI:** 10.3390/microorganisms14091934

**Published:** 2026-09-01

**Authors:** Muqadas Altaf, Waseem Khalid, Zhijia Fang, Haroon Munir, Afifa Aziz, Muhammad Bilal Hussain, Qingping Wu, Ravi Gooneratne

**Affiliations:** 1 College of Food Science and Technology, Guangdong Provincial Key Laboratory of Aquatic Product Processing and Safety, Guangdong Provincial Engineering Technology, Research Center of Marine Food, Key Laboratory of Advanced Processing of Aquatic Products of Guangdong Higher Education Institution, Guangdong Ocean University, Zhanjiang 524088, China; muqadasaltaf304@gmail.com (M.A.); afifaaziz44@gmail.com (A.A.); 2 Department of Food Sciences, Government College University Faisalabad, Faisalabad 38000, Pakistan; haroon.munir@gcuf.edu.pk (H.M.); mbilalhussain@gcuf.edu.pk (M.B.H.); 3 Department of Inorganic, Organic and Biochemistry, Faculty of Chemical Sciences and Technologies, University of Castilla-La Mancha, 13071 Ciudad Real, Spain; waseem.khalid@uclm.es; 4 School of Biology and Biological Engineering, South China University of Technology, Guangzhou 510641, China; 5 Department of Wine, Food and Molecular Biosciences, Lincoln University, Lincoln 7647, Canterbury, New Zealand; ravi.gooneratne@lincoln.ac.nz

**Keywords:** phage recognition, intracellular defense, adsorption kinetics, reciprocal adaptation

## Abstract

The widespread exposure of multidrug-resistant bacterial strains due to excessive use of antibiotics has accelerated the bacteriophage applications as highly specific antibacterial agents competent to target resistant pathogenic *Vibrio* through receptor-mediated adsorption and lytic infection. *Vibrio* pathogens are responsible for substantial economic losses, environmental instability, and foodborne infections in humans through contaminated seafood and water. However, the molecular determinants remain incompletely understood, commanding the *Vibrio*-phage host specificity, host range, and resistance evolution. This review highlights the comprehensive overview of structural, and functional framework of *Vibrio* surface receptors responsible for phage recognition, including outer membrane vesicles (OMVs), porins, lipopolysaccharides (LPS), capsular polysaccharides (CPS), flagella, and pili along with specialized bacteriophage tail spike proteins known as receptor-binding proteins (RBPs). We also explored the mechanisms against phage-mediated lysis, including receptor modification, intracellular defense systems, physiological remodeling, and structural adaptations that regulate burst size, lysis timing, and resistance evolution with emphasis on significant progress in phage biology and therapeutic development. It is critical to grasp the molecular-level interaction mechanisms due to limited marine phage ecological data, lack of standardized resistance databases, and regulatory constraints. Future perspectives emphasize the engineered phages, multi-omics integration, and artificial intelligence-driven phage design for the sustainable management of *Vibrio*sis and improved aquaculture biosecurity.

## 1. Introduction

The *Vibrio* genus is the leading bacterial group affecting the aquaculture sector at larval stages. *Vibrio* infections are passed on to humans via infected seafood and *Vibrio*-contaminated water. Reared fish in the Mediterranean climatic zone known as gilthead sea bream (*Sparus aurata*) has been widely infected with *Vibrio*. This valuable fish is a source of transfer of *Vibrio* infection to human beings [1]. The elements resulting in a high pathogenic bacterial load in larva-culture stations include artificial ecosystems, fish feed, fish hosts, organic materials from animal excreta, and rearing water [2]. The investigation of the causative agents for foodborne diseases and subsequent development of preventive steps to control the outbreak and related economic loss is the basic goal and priority of a rational food safety program. The major causative agent of foodborne illness linked to raw or undercooked seafood and shellfish is enteropathogenic *Vibrio*. These are entero-pathogenic *Vibrio* spp., which are Gram-negative bacteria found in estuarine ecosystems [3].

The pathogenic species of the *Vibrio* genus are *V. splendidus*, *V. cholerae*, *V. campbellii*, *V. vulnificus*, *V. mimicus*, *V. parahaemolyticus*, *V. alginolyticus,* and *V. harveyi* [4]. A combination of climate, population, and socioeconomic projections into a new generation model that is the Coupled Model Intercomparison Project 6 Framework showed that *Vibrio* could inhabit 38,000 km of the contemporary coastal area by the year 2100. Moderate challenges between 1980 and 2020 bring up a doubling of the number of units from 610 million to 1100 million, and this can reach 1300 million by 2025 [5]. Across a theoretical symbiome-pathobiome continuum, three communal factors such as microbe-microbe, host-microbe, and environment-microbe interactive forces conclude the aquaculture microbiome’s final status. In order to determine the molecular mechanisms involved in the persistence, host colonization, and disease development by dynamic microorganisms, it is a prerequisite to improve the list of host-related microbes that are able to be cultivated under specific conditions. For example, the functional parameters of pathobiomes and symbiomes are known for juveniles and fish larvae/live feed, making it much more feasible to manage the disease development in aquaculture systems and maintain consistency [6].

Phages are microbiological entities that reduce the *Vibrio* load in the targeted systems without affecting the host microbiota [7]. Lytic phages have substantial preventive action against *V. parahaemolyticus*, *V. harveyi,* and *V. alginolyticus* [8,9,10]. Phage cocktails exhibit strong lytic efficiency in *Vibrio* killing by using phage-derived enzymes (e.g., endolysins and holins) that degrade the peptidoglycan structures of the *Vibrio* envelope [11]. Although the phages are effective, there are resistance mechanisms that need to be elaborated to assist the phage efficiency decline. The phage adsorption is badly affected due to receptor-level modifications, restriction-modification enzymes, and CRISPR-Cas adaptive immune systems [12]. Quorum sensing regulates the biofilm formation that protects the *Vibrio* from phage adsorption [13]. A comprehensive record of recent studies shows that there is a lack of extensive or systematic information about the structural receptors on the *Vibrio* envelope involved in phage adsorption that counts the phage lytic efficacy [14]. Phage receptors like LPS, CPS, type IV pili, flagella, and OMPs modulate the phage adsorption, and quorum sensing confirms that CPS phage receptors increase the phage adsorption on the V, alginolyticus cell envelope [15]. Mutations in O-antigen gene clusters also affect the phage lytic efficiency [16]. Phages, being an alternative to antibiotics, have a potential role in *Vibrio* inhibition strategies. The insufficient understanding of the phages and lack of knowledge on systematic identification, implication, and characterization of the receptors and host adaptive modification have created problems for the smooth and sustainable phage therapy.

Diverse and representative collections of phages from a variety of sources are required to study the biology of host populations; the habitat, density, and genetic diversity of the hosts are therefore important variables in phage ecology. A study compared two populations of bacteria and their phages from an oyster farm at the same site as part of a time series sampling program. The variety of oligomannose structures of the O-antigens is directly related to the host range of the phage, since the tail spikes and tail fibers are evolved to recognize specific oligomannosidic structures on the O-antigens on the *Vibrio* outer membrane [17]. Receptor accessibility is strongly dependent on the physiological state of the bacterial host. For example, the availability of receptors on bacteria in the biofilm could differ from that on planktonic bacteria, altering the infectivity of the phages. One such example is that in *V. parahaemolyticus*, a quorum sensing (QS) system is present. The homolog of QS receptor-transcription factor VqmA is found in phage VP882 and is a receptor for the *Vibrio* QS autoinducer DPO. When DPO is bound by phage VqmA, transcription of an anti-repressor in the phage allows lytic infection at high host-cell densities. However, the bacterium can escape from the action of the phages if the host’s QS activity is naturally silenced, preventing the recognition [18]. There is a lack of a systematic explanation in literature of how *Vibrio* makes itself adaptable under different conditions when it comes under phage attack. The unclear apprehension of host-phage systems, several physical and structural modifications, and the influence of intracellular adaptations need to be mapped out to solve these problems.

The objective of this study is to elucidate the underlying mechanisms involved in phage and *Vibrio* interactions. It also characterizes the primary structural receptors on the *Vibrio* outer membrane that affect the phage lysis efficiency. The characteristic functions of RBPs, OMVs, porins, LPS, CPS, flagella, and pili that determine the phage specificity and lysis efficiency have been evaluated, while highlighting the diverse host adaptation mechanisms, including receptor modifications, intracellular defense systems, lysis gene mutations, physiological remodeling, and structural adaptations that contribute to phage resistance.

## 2. Accessibility of Primary Phage Receptors

Receptor accessibility involves the presence, exposure, and functional availability of receptors on the bacterial surface, which are dynamic and can be influenced by a variety of bacterial strategies and environmental conditions. The RBPs are variable and can be recombined, enabling them to adapt their host range. In the case of *Vibrio*, these receptors may be a part of the lipopolysaccharide (LPS), outer membrane proteins, and flagella. To adapt the diversity of hosts, both specialist and generalist phages must be evolvable, which depends on the evolvability of the RBPs. Modification of RBPs has been shown to be a way to modify or expand a phage’s host range, which provides new opportunities to use phages as therapeutic agents against difficult pathogens. The specific lytic activity of *Vibrio* phages is useful for targeted therapy, but it also poses problems because there is a large diversity among the phages and resistance can develop in the bacteria. This requires an accurate understanding of the molecular dynamics of phage-hosts interactions. There are advanced computational and deep learning approaches being developed to characterize phages and to predict their host range to inform the design of engineered phages that are more specific and effective. This ongoing coevolution process between phages and their bacterial hosts in nature further reinforces this process of specificity and adaptation [19].

The outer membrane of Gram-negative bacteria, such as *Vibrio* species, is an asymmetric membrane made up of lipopolysaccharides, phospholipids, lipoproteins, and integral OMPs [14,20], Table 1.

### 2.1. Receptor-Binding Proteins (RBPs)

**Figure 1:**
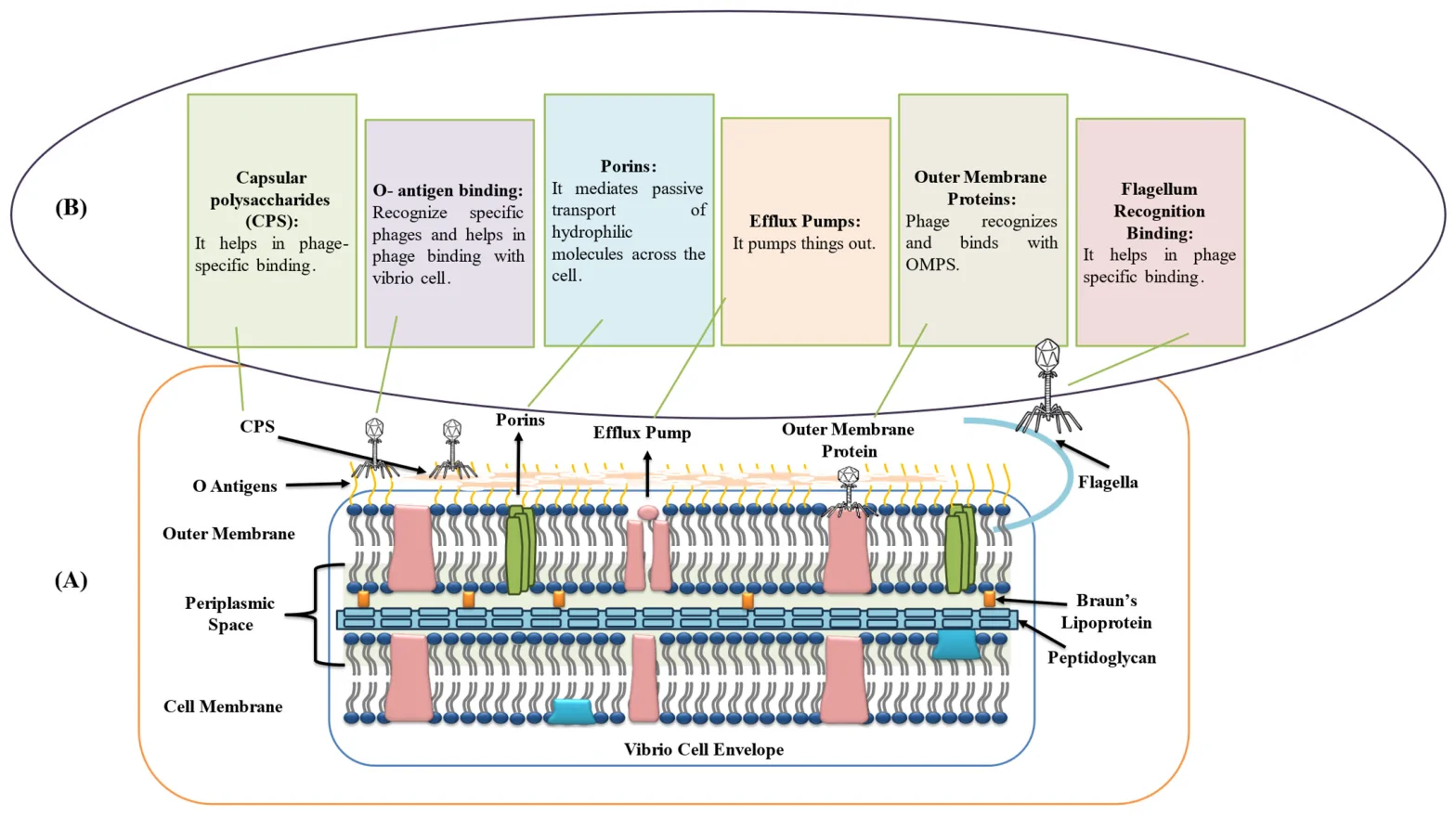
Accessibility of Primary Phage Receptors. (**A**) Structure of *Vibrio* Envelope and (**B**) Phage Receptor Binding Sites.

Different *Vibrio* phages have been identified with their lytic effect and specificity towards pathogenic *Vibrio* strains such as *V. parahaemolyticus* and *V. alginolyticus*. The results of these studies are consistently strain-specific, with many *Vibrio* phages identified as potential candidates for aquaculture and clinical-specific biocontrol. For example, *Vibrio*-specific lytic phages such as *Vibrio* phage KIT04 target *V. parahaemolyticus*, and other *Vibrio* phages, such as TDD and SRI, target *V. alginolyticus*. Continuous research is also under way for the discovery of new phages that have specific lytic activity against environmental *Vibrio* strains such as cholera-producing *Vibrio* species, with promising results. High and precise targeting by *Vibrio* phages allows them to efficiently infect bacterial hosts, and the accessibility of primary phage receptors is a key factor determining high lytic specificity and precision targeting. For the bacteriophages, the initiating step of infection involves the recognition and binding of specific surface structures on their host bacteria by receptor-binding proteins (RBPs), which include tail fibers and tail spikes, as is the case with the viruses infecting *Vibrio* species. In the other, a phage (BUCT549) infects *V. alginolyticus* and recognizes its flagella. The outer membrane protein EpsD or a polyQ protein called VcpQ are in examples of a receptors for *V. cholerae* phages [30]. Figure 1 shows the phage receptors on the *Vibrio* membrane.

Many phages are narrow host range phages that only infect specific strains of a bacterial species, while some phages, e.g., ones in the genus *Schizotequavirus*, have wide host ranges that infect several species of the genus *Vibrio* [18]. The broad host range phages frequently exhibit adaptive genomic plasticity and complicated infection strategies, yet the underlying mechanism still depends on specific interactions of their RBPs, but with more versatility.

The interaction between a bacterium and a phage is a major factor in the evolution and diversity of bacteria and can thus strongly influence the genetic and functional dynamics of bacterial communities. In this study, it has been revealed that quorum sensing (bacterial cell-cell communication) is an important, but so far not widely recognized, factor that affects the outcome of a phage-bacterial encounter in the fish pathogen *V. anguillarum*. In particular, the mutant cells of *V. anguillarum* (ΔvanT) are in a low-cell-density state and hyper-express OmpK, a phage receptor, and are very susceptible to phage KVP40 but become protected from phage infection by their enhanced biofilm phenotype. In contrast, cells fixed in the high cell density state (ΔvanO mutant) are nearly impervious, because the quorum-sensing system downregulates OmpK in these cells. The phenotypes of the two quorum-sensing mutant strains reflect the behavior of the wild-type *V. anguillarum*; the expression of the gene ompK is higher in aggregated cells than in free-living cells within the same culture, the number of bacterial cells expressing ompK is inversely correlated with the level of N-acylhomoserine lactone quorum-sensing signal in the culture medium, and *V. anguillarum* can defy phages with only one of these two mechanisms, and this is the dominant mechanism. Our results, combined, show that *V. anguillarum* uses quorum-sensing information to select between two alternate antiphage defense strategies. In addition, the high level of non-mutational defense mechanisms in strain PF430-3 indicates that it has adapted to cope with the KVP40 phage very flexibly, perhaps permitting the permanent coexistence of phage and host [31].

One of the basic issues in developing an accurate immunodiagnostic assay and a multivalent vaccine of pathogenic *Vibrio* species in food and aquaculture is identification of outer membrane proteins (OMPs) that have the desirable specificity and surface availability. A total of 4831 non-redundant proteins of *V. parahaemolyticus* were systemically screened for bioinformatical prediction of the signaling peptides, the transmembrane (TM) α-helix, and the subcellular localization; with the use of bioinformatics, 101 OMPs were selected. The sequence homology analysis with 32 *Vibrio* spp. and all non-*Vibrio* strains showed that 15 OMPs were conserved in at least 23 *Vibrio* spp., including BamA (VP2310), GspD (VP0133), Tolc (VP0425), OmpK (VP2362), OmpW (VPA0096), LptD (VP0339), Pal (VP1061), flagellar L-ring protein (VP0782), flagellar protein MotY (VP2111), hypothetical protein (VP1713), fimbrial assembly protein (VP2746), VacJ lipoprotein (VP2214), agglutination protein (VP1634), and lipoprotein (VP1267); high adhesion probability of flgH, LptD, OmpK, and OmpW indicated that they were potential multivalent *Vibrio* vaccine candidates. At least one of two OMPs was relatively common among *Vibrio* spp., while 19 OMPs were relatively found in *V. parahaemolyticus,* including OmpA-like protein (VPA073), CsuD (VPA1504), and MtrC (VP1220). By enzymatically shaving the cells, the authors found the capsular polysaccharides were most likely the limiting factor in protease action, and the glycosidases enhanced trypsin accessibility to OMPs. The OmpA (VPA1186, VPA0248, VP0764), Omp (VPA0166), OmpU (VP2467), BamA (VP2310), TolC (VP0425), GspD (VP0133), OmpK (VP2362), lpp (VPA1469), Pal (VP1061), agglutination protein (VP1634), and putative iron (III) compound receptor (VPA1435) have better availability on the cell surface. The protein sequences of *V. parahaemolyticus* were screened comprehensively for their OMPs using the integrated bioinformatics predication methods, which resulted in 4831 sequences. The overall homology analysis of the other bacteria through the Basic Local Alignment Search Tool (BLAST, 1) was used to obtain the differential OMPs and vaccine candidates of *V. parahaemolyticus* and *Vibrio* spp using the online server TMHMM 2.0 and HMMTOP (also known as Community College Transfer Opportunity Program [CCTOP]. The adhesion probability analysis was used to further select the possible vaccine candidates [32]. The surface proteome of *V. parahaemolyticus* was subjected to enzymatic shaving (glycosidases, trypsin) of intact cells and mass spectrometry identification of the peptides thus obtained to experimentally study the OMPs.

The OMPs of *Vibrio* spp. play an important role in a variety of processes such as bacteriophage receptors. Phages do not have OMPs but instead use bacterial OMPs for attachment and subsequent infection, playing a critical role in phage host range and infection efficiency. Phages are viruses that require specific interaction with host receptors for effective infection. The interaction typically includes receptor binding proteins present on the phage surface; for instance, the OMP receptors are recognized by the *Straboviridae* phages by specific extracellular loops of OMPs. *V. parahaemolyticus* 1010 (RIMD 2210001) has a gene named ompK with 789 nucleotides and 263 amino acids. The mature OmpK protein, with the removal of a signal peptide with 20 amino acids, is estimated to have a molecular mass of 27,458 Da [33]. A number of OMPs have been found in *V. cholerae* and *V. parahaemolyticus,* which are involved in phage recognition, among which OmpU and OmpK are most studied. The interaction between *Vibrio* and its phages is also complex, with other OMPs, such as OmpW, OmpA, and OmpS, also playing a role. Another OMP that functions as a receptor for typing phage VP5 of *V. cholerae* O1 El Tor biotype is ompW. An 11-bp deletion in OmpW has been associated with VP5 resistance among epidemic strains and could be used as a marker for cholera surveillance [34].

OmpK is also reported as a common antigen in the *Vibrio* species and has been investigated as a vaccine against *Vibrio* infections in fish such as *V. harveyi*, *V. alginolyticus,* and *V. parahaemolyticus* [35]. The OmpU outer membrane protein is part of the ToxR regulon of *V. cholerae* and was recently shown to be a possible adherence factor for this species. The gene coding for OmpU has been cloned and sequenced using PCR and degenerate oligonucleotide primers based on the internal peptide sequence of the purified OmpU. The ompU gene is predicted to encode a 36,646 Da protein, which is present in both cholera toxin-positive and -negative *V. cholerae* O1 and O139 strains. OmpU is another major OMP, which is mainly associated with *V. cholerae*. OmpU is regulated by the ToxR regulon, a master regulatory network that regulates expression of virulence genes in *V. cholerae* in response to environmental signals in a host [36].

The ToxR regulon also regulates expression of virulence factors such as Toxin-Coregulated Pilus (TCP), which is a receptor for the CTXϕ bacteriophage. The main receptor for the CTXϕ phage is the TCP pilus [37].

Hemocyanin may be able to negatively regulate the metabolic process of *V. parahaemolyticus*. Hemocyanin is a multifunctional protein found in arthropods and mollusks that has multiple functions in immune defense, such as antibacterial activity, as well as functions in oxygen transport. The molecular mechanism of pathogen recognition and antibacterial activity by hemocyanin is still poorly understood; however, *V. parahaemolyticus* OMPs binding to Litopenaeus vannamei hemocyanin were isolated using the pull-down assay in the present study. Two interacting OMP bands were identified as OmpC and OmpU, and the heterogeneous interaction between hemocyanin and two OMPs was additionally confirmed by far-Western blot. The ompC and ompU deletion mutants were constructed, and we observed that the agglutinating activity and antibacterial activity of hemocyanin were greatly reduced in comparison to the wild-type strain [38,39]. Hemocyanin treatment resulted in four intracellular proteins of *V. parahaemolyticus*; fructose-bisphosphate aldolase and ribosomes were identified to interact with rOmpC and rOmpU, respectively. In addition, hemocyanin treatment caused a significant reduction in the mRNA expression levels of ompC, ompU, fbaA, rpsB, and rpsC. These results showed that OmpC and OmpU are the main targets of *L. vannamei* hemocyanin for pathogen binding and mediation of its antibacterial activities [40].

These OMPs serve as gatekeepers and regulate nutrient and molecule entry to the cytoplasm as well as interactions with the outside environment, including phages and host immune systems. The OMPs play an important role in bacterial survival and virulence as adhesins, porins, and components of secretion systems. Changes in these OMP receptors may lead to phage resistance, as the phage cannot attach to the receptor. A large proportion of the porins are trimeric in the outer membrane. They are highly expressed on the bacterial surface and thus have a number of important functions in host-bacteria interaction. These include porins that contribute to adhesion and virulence mechanisms involved in the pathogenesis and resistance of the bacteria to the antimicrobial substances [41].

### 2.2. Outer Membrane Vesicles (OMVs)-Mediated Defense Mechanism

Gram-negative bacteria also secrete outer membrane vesicles (OMVs), which may contain OMPs. These OMVs have different functions such as decoys or weapons against bacteriophages for better bacteria defense against bacteriophages [42]. Bacterial OMVs are multifunctional and released by both Gram-negative and Gram-positive bacteria and are an integral part of microbial ecology and pathogenesis. They serve roles in biofilm formation, nutrient uptake and cell-to-cell communication in microbial communities, host-microbe interactions, and immune modulation in the host. Bacteria are able to adapt and survive in other environments through the use of EMVs as detergents, mediators of biochemical cycles, and defense systems. EMVs carry immunomodulatory molecules that disrupt host immune signaling pathways so that bacteria can escape immune recognition [43]. The presence of OmpU in both OMVs and the biofilm matrix thus suggests that it is a multifunctional Omp that could function in vesicle-mediated processes, biofilm architecture, and bacterial adhesion. The outer cell membrane and the surface of the vesicles are coated with the same receptors (such as LPSs or OMPs) as the OMVs, so binding of the phage to OMVs will (but does not always) neutralize the phage. To mimic the OMVs blocking function of Gram-negative bacteria, these vesicles must contain receptors that are the main recognition sites for phages [44]. OmpU is an outer membrane protein that mediates the bacterial interaction and helps in the initial attachment of phage required for biofilm establishment. After the attachment occurs, the other matrix components like RbmA, RbmC, Bap1, and VPS make a strong cell-surface and cell-cell interaction to promote the biofilm maturation [45]. Therefore, OmpU is linked to biofilm formation via its potential activity involved in the initial adsorption stage, and the presence of OMVs highlights its potential relevance to the host interaction and the phage recognition. After the initial attachment has occurred, the matrix components and the VPS promote the cell-cell introductions and help in the cell surface adhesion with different efficiencies. The VPS makes strong adhesion, while Bap1 makes weak adhesion [46]. Thus, OmpU gives a crucial interface between the cell envelope and its surrounding matrix, facilitating the attachment that enables matrix accumulation and biofilm maturation. Coral-associated bacterial OMVs provide a protective effect against phage infection, and this effect was compromised by increased temperature. These complex relationships are cryptic and may be involved in structuring coral microbiomes under varying environmental conditions. Moreover, the OMVs secreted by bacteria could interact with phages, potentially influencing the direct and indirect impact of viruses (including phages) on coral reefs. It demonstrates the importance of the ability of bacteria to produce OMVs as a defense mechanism, which should be considered in the use of phage therapy or prophylaxis in coral disease treatments [47]. The functions of OMVs in rendering phages more sensitive have been examined in a few studies, especially regarding the role of phages in combating the resistance of bacteria to phage infection. One such example is that OMVs of pathogenic *Vibrio* cholerae strains can dose- and receptor-dependently neutralize virulent phages, thus limiting the predation of phages. From this observation it is suggested that OMVs could serve as decoys, binding to phages and preventing their infection of the bacterial cells [48]. Another mechanism is the outer membrane vesicles (OMVs) that have a critical function in stimulating and distributing QS molecules. It is likely that the delivery of QS molecules to the target cells via OMVs by Gram-negative bacteria is more prevalent in marine environments than previously reported. Not only does this delivery system prevent degradation of the signal during transit, but it also allows receptor-mediated delivery of the signal to specific sites. Given the large number of hosts impacted by QS and the importance of QS signals in aquatic environments, this is an important area for future study that will help clarify the role of QS signals in interkingdom interactions in the marine environment [49].

### 2.3. Outer Membrane Porins-Mediated Cellular Defense

The outer membrane proteins in Gram-negative bacteria, termed as porins, are involved in the transport of hydrophilic molecules like nutrients, ions, and antimicrobials across the outer membrane. The outer membrane of Gram-negative bacteria encapsulates the plasma membrane, thereby protecting it from the harsh external environment. Because of the special membrane-spanning proteins called PORINS, this membrane is a sieving barrier. These porins are β-barrel channel proteins that transport hydrophilic molecules passively and exclude large and charged molecules. They also act as receptors for phage and complement proteins and are involved in modulating the host cellular responses. Besides, the use of porins as adjuvants, vaccine candidates, therapeutic targets, and biomarkers has gained attention [50]. This is a structural feature that is typical for porins, which are used for transporting proteins through the outer membrane [51]. These β-barrel channel proteins are formed by trimers and create a sieving barrier, which is essential for survival, and adaptation of bacteria in their environment [52]. Porins are important for bacteriophage-*Vibrio* interactions and subsequent host lysis in several aspects, such as phage adsorption and the release of progeny virions. The first step in bacteriophage infection is the adsorption of a phage through specific phage-receptor-binding protein (RBP) and bacterial surface receptor interactions. Some phages target parts of the lipopolysaccharide (LPS) as primary receptors, while others bind directly to outer membrane proteins (OMPs) [53]. A particular porin gene (Vp-porin) has been isolated in *V. parahaemolyticus*; the gene has been found to be very conserved among *Vibrio* spp. The 3D structure of Vp-Porin predicted by AI suggests a transmembrane β-barrel domain with 20 strands [54].

The porin permeability characteristics are important as they affect the sensitivity of bacteria to antibiotics and other external factors since they allow or restrict entry into the cell. In general, the lytic pathway of phage involves a set of phage lysis proteins that penetrate the three envelopes of the Gram-negative bacterium: the outer membrane, peptidoglycan, and inner membrane [55]. For example, holins permeabilize the cytoplasmic membrane, endolysins disrupt the peptidoglycan layer, and spanins are involved in outer membrane disruption [56,57]. Some bacteriophages with a lytic phenotype contain the Rz/Rz1 protein system specifically targeting the external membrane of the host bacterium. Although porins are not necessarily the main pathways for phage DNA injection (which occurs via the tail structure their presence and activity can have an indirect effect on the efficiency of this step and the subsequent lysis [58]. The baseplate structure is complex; in tailed phages it directly participates in the recognition of the host and penetration of the cell wall. This initial binding by tail fibers causes conformational changes in the phage, making the binding irreversible, and phage DNA is injected [59]. The phage then hijacks the host cell’s machinery for synthesis and replication of new virions, and the cell eventually bursts open, releasing its progeny virions. Various molecules require a permeability mediated by porins to get in. In the case of Acinetobacter baumannii, the antimicrobial resistance is related to the porin OprD. In the lytic cycle, after the enzymes (endolysins) that target the peptidoglycan and create pores in the inner membrane disrupt the internal components of the cell, the outer membrane must still be broken for the virion to be released. In this case porins may have a facilitative role in addition to the phage-encoded spanins or other disrupters of the outer membrane [60]. The proteins are presumably involved in concert in the destabilization of the outer membrane, which facilitates the efficient release of newly formed phage particles. The end result of the lysis process is the release of progeny virions into the environment to start new infection cycles [61]. In conclusion, porins are indispensable channels of the outer membrane of *Vibrio* and other Gram-negative bacteria, which directly affect the bacterial physiology and indirectly affect the interaction with bacteriophages. The adsorption of phage is mostly mediated by specific RBPs, but some porins may serve as direct receptors for phages. In addition, the overall integrity and permeability of the outer membrane, which is influenced primarily by porins, may influence the entry of phage and the efficiency of the lytic cycle, especially during the release of phage progeny. A biological arms race between bacteria and phages can result in the modification of porins as a defense mechanism by bacteria and adaptation to those changes by phages.

### 2.4. The Lipopolysaccharide (LPS) and Capsular Polysaccharide (CPS)

*Vibrio* is known to develop a number of strategies to alter the accessibility of receptors and to escape recognition by phages. These include the regulation of the expression of receptors and phase variation of surface antigens. In addition to O-antigens, other LPS components may serve as primary receptors (core LPS). In the case of Rhizobium etli, mutations in genes required for the synthesis of LPS, such as wreU and wreV, can confer resistance to the phage infection, highlighting the importance of LPS in phage adsorption and infection [19]. This suggests similar genetic changes in that *Vibrio* spp. may impact their sensitivity to the phage lysis.

**Figure 2:**
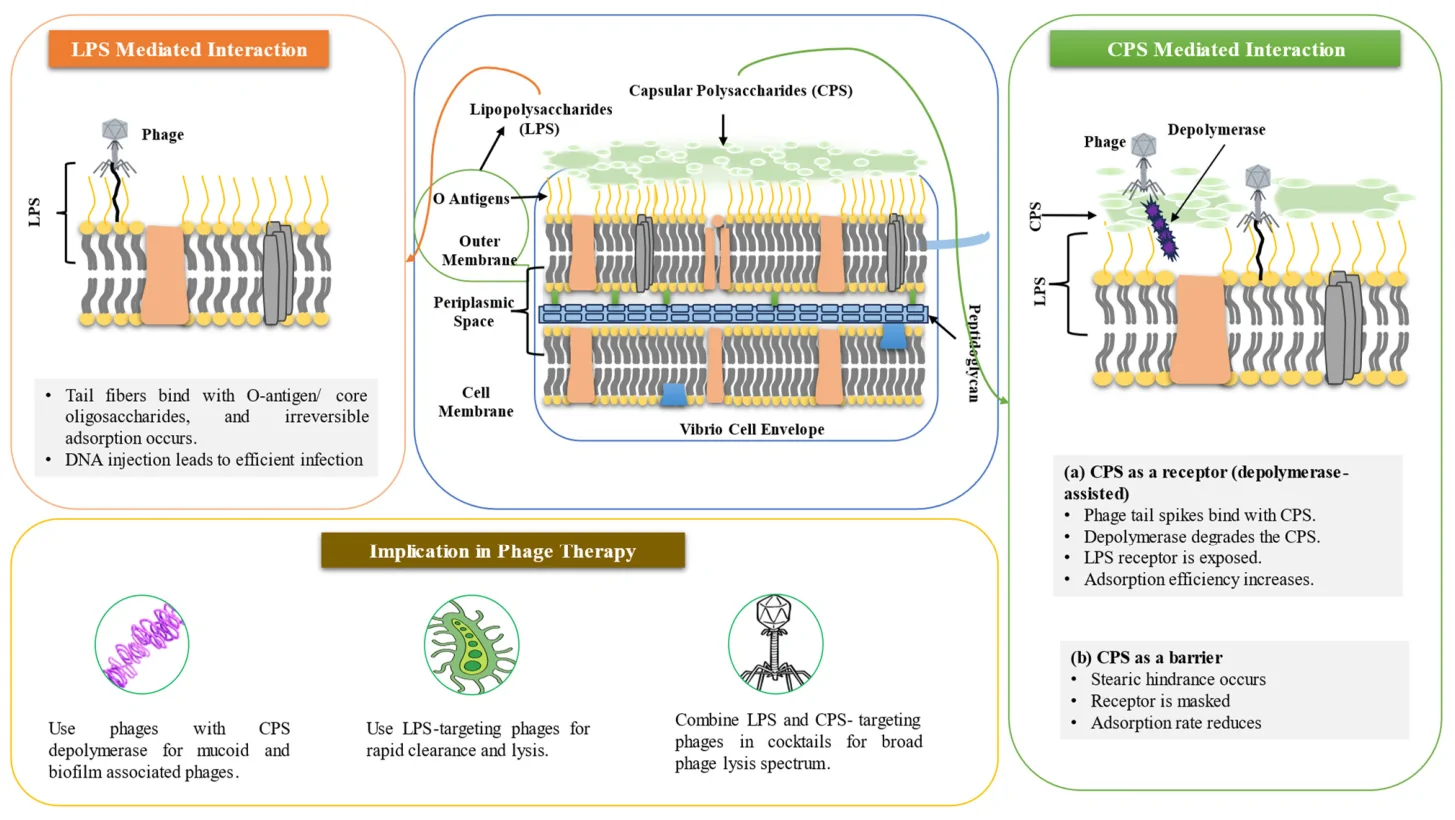
LPS-Mediated interaction and CPS-Mediated interaction between Phage and *Vibrio*.

*V. parahaemolyticus* is one of the most significant foodborne pathogenic bacteria that poses a serious threat to aquaculture and public health; thus, there is a need for alternative biocontrol approaches like phage therapy. A study on vB_VpM-PP270 revealed the mechanism of action of this phage against *V. parahaemolyticus* VP270. The study identified capsular polysaccharide (CPS) as the main receptor for the phage adsorption and a tail spike protein, ORF22, as the receptor binding protein. PP270 is stable over a broad pH range and temperatures and has excellent growth inhibitory properties on chilled shrimp and in culture for VP270. The phage-resistant mutants have mutations in the genes involved in CPS biosynthesis that inhibit the adsorption of phages and facilitate the formation of biofilm. The results offer insights into phage-host interactions and could serve as a blueprint for creating phage-based treatments that are less susceptible to resistance development, which may be important for food safety and sustainable aquaculture practices. However, co-incubation with VP270-derived CPS markedly decreased the phage adsorption rates compared to VP255-derived CPS, which was similar to PBS controls, suggesting that VP270 CPS competes with phages for the same binding site on the host. Combining this evidence, namely, the carbohydrate nature of the PP270 receptor, the localization of the resistance mutations (ugd, gtr, and gtr2) to the CPS cluster, the reversion of the resistances in deletion mutants by complementation with an active copy of the gene, and the adsorption inhibition by the host CPS, we propose that CPS is the main adsorption receptor for the phage PP270. Key findings indicate that PP270 is a new species in the class *Caudoviricetes*, which enters VP270 through a specific recognition of CPS by the tail spike protein ORF22. Resistance involves mutations in the genes involved in CPS biosynthesis (ugd, gtr, gtr2) that change surface features and enhance biofilm production in the host. To understand the functions of ugd, gtr, and gtr2 in resistance, we constructed deletion strains (Δugd, Δgtr, and Δgtr2) and complemented strains (Δugd: ugd, Δgtr: gtr, and Δgtr2: gtr2). Deletion mutants were resistant to PP270 as revealed by spot assays, while complemented strains were able to form plaques similar to the wild type, indicating that these genes affect phage susceptibility. The Δugd phenotype was dry and rough (TR) in comparison to the smooth and moist (OP) of the wild-type VP270, while the Δgtr and Δgtr2 phenotypes were intermediate; complementation resulted in the expression of the wild-type OP phenotype. Deletion mutant colonies showed less of the red-staining area (decreased production of polysaccharide) that was restored in complemented colonies, indicating a role in the production of extracellular carbohydrate [21]. A correlation between gene function and biosynthesis of receptors was shown by adsorption assays, which showed that the deletion mutants exhibited reduced rates that were restored by complete mutation. All the deletion mutants were found to have an enhanced ability to form biofilm, and the ability to form biofilm was restored in the complemented strains. Figure 2 shows the phage interaction with LPS and CPS.

### 2.5. Flagella and Pili

It has long been known that in *Vibrio* phage interactions the role of flagella and pili in the initial stages of infection, attachment (adsorption), is not directly related to the subsequent lytic stage of the infection process. These surface appendages play an important role in determining host specificity and adsorption efficiency and are crucial for the evolutionary dynamics between *Vibrio* species and their phages. Understanding these mechanisms involved is critical to develop effective detection systems and therapeutic strategies against pathogenic *Vibrio* like *V. cholerae*, *V. parahaemolyticus,* and *V. alginolyticus.*

The flagellum, especially the sheathed polar flagellum of many *Vibrio* species, is a primary receptor site for an important group of phages termed flagellotropic phages. The well-studied phage ICP1, which infects *V. cholerae*, specifically binds to the flagellar sheath through its tail fiber proteins. This interaction causes an induced conformational change in the phage that eases the injection of the phage DNA into the host’s cytoplasm [62,63]. Mutations in bacteria that affect flagellar assembly or motility can confer resistance, but at a considerable fitness cost, due to the importance of flagella to the bacteria. Flagellar motility is important for chemotaxis, biofilm formation, and colonization ability, which are essential for virulence and environmental fitness [64,65]. In recent studies, flagella-mediated adsorption of the bacteria has been shown to be not only passive but also the hydrodynamic flow generated by swimming bacteria can increase phage encounter rates, indicating that there is a complex relationship between bacterial behavior and phage predation [66].

In addition to the receptors for virulence factors, pili are used by other *Vibrio* phages as alternative/secondary receptors, particularly type IV pili (T4P) and mannose-sensitive hemagglutinin (MSHA) pili. The phage VP2 and VP5 use the MSHA pilus for initial attachment, which binds to the MSHA pilus depending on the specificity of pilin subunit composition and genes for biogenesis (e.g., the mshA–mshL genes) [62]. Of note, a few phages have been found to use more than one receptor because for some Campylobacter *jejuni* phages, such as model phage F341, pili, in addition to other surface molecules like lipo-oligosaccharides, are required for productive infection [67]. The retraction dynamics of T4P can also affect infection outcome: some RNA phages bind to pili and cause them to leave the bacteria, thus immobilizing the bacterium and allowing the virus to enter. The double effect, in addition to internalizing the phage, is to disrupt the function of the host, underlining the sophistication of co-evolutionary adaptations in phage-pilus interactions [59].

Importantly, the role of flagella or pili in the lysis stage of the phage life cycle is not directly involved. Enzymatic machinery encoded by the phage (holins and endolysins in the case of Gram-positive, spanins in the case of Gram-negative) is responsible for lysis. The flagella and pili are thus essential in initiating infection, but the termination process is completely dependent on the lytic proteins expressed intracellularly. A multidisciplinary toolkit is used for the detection of phage-flagella/pili interactions. Direct visualization of phages bound to flagella or pili using electron microscopy, especially negative-stain transmission electron microscopy (TEM) and cryo-electron tomography (cryo-ET), can be used to examine phages bound to flagella or pili, revealing the binding geometry and stoichiometry. Functional assays include adsorption inhibition with anti-flagellin or anti-pilin antibodies that inhibit the binding of phages and decrease plaque formation. So are genetic methods, which involve making flagellar (flaA) or pilus (mshA) mutants of the same strain and testing them for susceptibility to wild-type strains to confirm receptor identity [28]. Further, the purification of receptor-binding protein (RBP) and subsequent binding affinity measurement by surface plasmon resonance or by microscale thermophoresis can quantify binding affinities to isolated flagellin or pilin subunits [68].

Recent developments use structural biology to achieve atomic-level mechanisms. The structures of phage tail complexes bound to purified pili by cryo-EM have revealed ways of escaping the immune system and the determinants of receptor specificity [69]. Engineered reporter phages, which are specifically designed to need functional flagella or flagella/pili to infect, can be employed in rapid diagnostic assays to detect live pathogenic *Vibrio* cells in seafood or water samples in aquaculture settings. The detection platforms are highly specific and sensitive and capitalize on phages’ natural requirement for surface appendages.

To summarize, flagella and pili play a key role in the attachment of *Vibrio* phages, acting as ‘gatekeepers’ that are central to host range and co-evolutionary arms races. They are not involved in the lysis itself but only in adsorption, which is an enzymatically self-regulated process. Detection strategies take advantage of this type of molecular recognition, using imaging, genetic, biochemical, and biophysical approaches to dissect and exploit these interactions in basic research and applied biotechnology.

## 3. Detection Methods Used to Test Different Resistance Factors

The first line of defense is adsorption inhibition, which is the quickest defense mechanism that can be deployed. Works through receptor modification or loss: alters LPS structure, outer membrane proteins (e.g., OmpU), or capsule polysaccharides [16]. This is accomplished phenotypically by efficiency of plating (EOP) assays and adsorption kinetics as determined by time-resolved centrifugation or radiolabeling [70]. Using *V. parahaemolyticus* and *V. cholerae*, it has been genotypically detected by sequencing the entire genome (whole genome sequencing [WGS]) followed by identifying the variants in genes involved in LPS biosynthesis (such as the wbeT and rfb cluster genes), porins (such as the ompU and ompK genes), or capsule regulators [71,72]. Importantly, this resistance comes at a high pleiotropic cost: the loss of OmpU would also be expected to have negative consequences for iron acquisition, a cost that was directly observed in *V. alginolyticus*, where the phage-resistant mutants were significantly less competitive in iron-limited conditions [73]. Anti-OmpU antibodies disturbed the extracellular/intracellular iron ratio, decreasing the extracellular iron by 78% after OmpU disruption. Under iron limitation, the expression of ompU was significantly affected. A predicted Fur binding site is present in the ompU promoter. Electrophoretic mobility shift assays showed that Fur was able to bind to the ompU promoter without Fe2+ present, but Fe2+ blocked this binding. This is great evidence for an OmpU-iron homeostasis relationship, but it’s not proof that OmpU is directly involved with the transport of iron or the direct interaction with siderophore uptake machinery. Experimental evidence suggests that OmpU is involved in functional interactions with iron homeostasis and acquisition in *Vibrio*. Regarding regulation, OmpU is controlled by the iron-responsive regulator Fur in V. alginolyticus and is shown to affect genes related to *Vibrio*bactin metabolism, siderophore transport, and iron ion homeostasis in V. cholerae by transcriptomic analysis [74].

The second level is represented by intracellular innate immune systems such as restriction-modification (R-M) and abortive infection (Abi). R-M systems can be modified in such a way that they will cleave the foreign DNA they have not been programmed to recognize; the activity of an R-M system can be phenotypically determined by plaque morphology analysis (decreased plaque size or increased lysis inefficiency) and DNA digestion assays with the host extracts [75,76]. Genotypic detection involves sequencing of the entire genome and scanning for the presence of R-M gene clusters (such as hsdMSR and mrr) by bioinformatic methods, for example, with the help of REBASE. Long-read sequencing (e.g., Oxford Nanopore) is being used for genotypic assessment, which helps resolve the repetitive ON/OFF switches in promoter regions, while RNA-seq is used to quantify defense gene transcriptional dynamics [77]. When triggered by phages, Abi systems kill the infected cells while ensuring the survival of the uninfected ones, and their phenotypic effects are typically a dramatic drop in burst size and premature lysis in one-step growth curves, sometimes with population-level immunity like that observed in *Pectobacterium* with Type I-F CRISPR [78]. The first step in genomic identification is screening for toxin-antitoxin (TA) modules, pif homologs, or BREX loci, followed by functional knockout [79].

CRISPR-Cas is the third and most advanced level, also known as adaptive immunity. It is characterized by sequence-specific interference mediated by spacers from previous encounters with phages. Phenotypically, CRISPR-mediated resistance is characterized as complete inhibition of lytic development, which can be detected as the lack of plaques on plates after phage sample plating and the inability to detect early phage gene expression with qRT-PCR. Most importantly, the Type III CRISPR-Cas system in *Vibrio* is not a DNA-cleaving system but is an abortive infection system, which confers population-level protection even after the death of individual cells [80]. The genotypic detection is definitive: WGS will detect cas genes (variants) from CRISPR, and the spacer analysis will align resistances to viral sequences (e.g., ICP1, pVco-7, and phage genome) [81]. That *V. cholerae* phage ICP1 has a CRISPR-Cas system embedded in its own genome to degrade the bacterial product of the CRISPR-Cas system, and it is a truly remarkable case of recursive molecular arms racing [82].

Lastly, there can be resistance through mechanisms of epigenetic regulation and/or modulation of defense gene expression by quorum-sensing. The efficacy of phages in *Vibrio* is extremely sensitive to quorum-sensing signals from the host, and single-cell reporter assays are necessary, and time-series EOP measurements are required across different host cell densities for phenotypic detection [83]. This multi-layer design principle is one of them: resistance is not a costless business. Each mechanism carries a fitness cost (slower growth rate, decreased virulence, or metabolic burden) that influences the outcome of the ecology. The correlation between phage resistance and decreased colonization ability has been observed in *V. parahaemolyticus*, and the correlation between phage resistance and decreased motility/biofilm formation has been observed in *V. cholerae*. Such trade-offs are not laboratory artifacts but evolutionary facts, and resistance surveillance is not a diagnostic activity but a prediction of pathogen evolution.

Genotypic resistance of *Vibrio* spp. to bacteriophages is detected by a combination of molecular and genomic approaches, which are used to identify genetic determinants that interfere with the ability of the phages to infect the bacterium. These methods are based on whole genome sequencing (WGS), gene-specific analyses, comparative genomics, and functional annotation and have been validated in various studies of *V. cholerae*, *V. parahaemolyticus*, and *V. alginolyticus*. Another important genotypic screening is bioinformatic screening of innate immune systems. R-M systems recognize unmethylated foreign DNA by searching the genome for certain conserved sequences (e.g., hsdS, hsdM, and hsdR) (Fineran & Fin, 2019) EBASE [75]. Based on known Abi-associated genes (such as toxIN, pif, or BREX components), a homology search will identify abortive infection (Abi) systems that are able to trigger programmed cell death upon phage infection and thereby protect clonal populations [84]. *Vibrio* toxin-antitoxin modules and stress-response regulators are less well documented as Abi systems in *Vibrio* than in other genera; there is a potential involvement in this genus that requires further genomic investigation [77].

Another mechanism of genotypic resistance is due to phase-variable expression of surface receptors from repetitive tracts of DNA that can undergo slipped-strand mispairing. These hypervariable regions are more likely to be misassembled by short-read sequencing technologies but can be better resolved by long-read sequencing technologies, such as Oxford Nanopore or PacBio [70]. For instance, the phase variation of the biosynthesis genes of the capsule in C. jejuni (a model organism for Gram-negative phage resistance) provides transient resistance against the capsule-dependent phages; similar mechanisms are supposed to also occur in *Vibrio*, due to the high occurrence of LPS and capsule phase variation [16]. The genes for O1-antigen biosynthesis (manA and wbeL) are phase-variable, homopolymeric. The gene switching changed the expression of O1-antigen, and led to the formation of phage-resistant subpopulations in relation to the O1-specific phage ICP1. These phage variants were strongly selected in simulated aquatic microcosms by phage predation [85]. The ability of *Vibrio* to remodel its cell surface dynamically by capsular polysaccharide phase variation might also enable it to change its phage susceptibility. For *Vibrio* vulnificus, the Op–Tr colony variation with high and low CPS production was reversible. Although the growth rates of Tr variants were similar to Op variants, these variants formed more biofilm and had less swimming motility. The transcriptomic analysis of the genes differentially expressed between the two phenotypes revealed that the bacterial surface was extensively remodeled, including the downregulation of six CPS-associated genes, repression of the tad-2 pilus cluster, differential expression of nine genes associated with c-di-GMP, 28 putative regulators, and eight putative porins, all of which are associated with the bacterial surface. The mechanism of such remodeling may generate phenotypically heterogeneous populations with differential phage susceptibility in *Vibrio* and may be even more significant for other surface structures such as CPS. In this study, however, neither direct phage adsorption nor direct resistance assays were performed, and so the relationship between CPS phase variation and phage resistance has not been proven experimentally [86].

Comparative genomics further enhances detection by contrasting resistant and susceptible isolates. Mutations in regulatory genes linked to the regulation of membrane integrity and phage adsorption were identified through multigroup sequencing of *V. parahaemolyticus* mutants and wild-type strains. Likewise, RNA-seq could be used alongside DNA-level analysis and could identify a trend of RNA downregulation of phage receptor genes or RNA upregulation of defense operons under phage pressure. To summarize, the methods for genotypic detection of phage resistance in *Vibrio* include WGS, bioinformatic analysis of defense islands (CRISPR-Cas and R-M), comparative variant calling, spacer-phage matching, and specialized sequencing of repetitive or epigenetically-regulated loci. These methods can all be used to accurately characterize resistance determinants, allowing for ecological surveillance and the rational design of phage cocktail combinations that avoid common resistance pathways.

## 4. Adaptations of Host for Vibrio-Phage Lysis and Effects on Lysis Efficiency

### 4.1. Receptor-Level Adaptations

Firstly, receptor-level adaptations are the first and most common obstacle to infection. *Vibrio* species often alter the structure of lipopolysaccharide (LPS) O-antigen, outer membrane proteins (e.g., OmpU), or flagellar components, among which are attachment sites for myoviruses, like vB_VpaM_R20L, and podoviruses, such as phi 50-12 [87]. The study of *V. cholerae* revealed that bile-induced downregulation of OmpU and anaerobic stress decreased phage adsorption by more than 80%, directly prolonging the latent period and lowering the effective burst size, as there were fewer successful infections [88]. Importantly, such changes incur measurable fitness costs, such as a reduced capacity to colonize the intestine in *V. cholerae* upon O-antigen truncation and reduced motility and biofilm formation in *V. parahaemolyticus* upon flagellar loss [16].

Receptor-level adaptations are the most basic adaptation mechanism that *Vibrio* spp. can use to resist phage predation, and they can affect the first step of the phage infection cycle, the adsorption process, at its most fundamental level, which thus determines the efficiency of the phage lysis. The adaptations are structural or regulatory changes in the primary receptors on the cell surface that are involved in phage tail fibers or baseplate proteins. The *Vibrio* receptors that play an important role in marine *Vibrio* like *V. cholerae*, *V. parahaemolyticus*, and *V. alginolyticus* are lipopolysaccharide (LPS) O-antigens, outer membrane proteins (OmpU, especially), and flagellar components, all of which are dynamically regulated in response to the environment and phage pressure.

LPS modifications are also a general, inherited mechanism of resistance. Following exposure to lytic phage, *V. alginolyticus* selects for mutants that lack the O-antigen but have retained the core oligosaccharide structure or have a modified core structure. These changes block the ability of the phage receptor-binding proteins (RBPs) from recognizing and binding the phage. Such mutations come at a significant fitness cost,, however, as shortened LPS causes the outer membrane to be less intact, resulting in greater sensitivity to antimicrobial peptides and decreased competitive fitness in nutrient-poor marine environments [73]. These resistant strains are shown to have down-regulated expression of genes that help transport iron and amino acids, functions commonly associated with the action of LPS-associated porins, further reducing growth rates as shown by transcriptomic analyses.

The added difficulty comes with flagellar resistance. Some *Vibrio*-phages, e.g., those of *V. parahaemolyticus*, attach to the flagellar filament and/or motor proteins. Motility deletions (deletions in flaA or regulatory genes such as luxR) give complete resistance to these phages. However, the loss of flagella significantly affects the ability to form biofilms and to move in an aquatic environment by chemotaxis, which are essential for the colonization of biotic (such as shellfish, and coral) and abiotic surfaces. Interestingly, two myoviruses with >95% genome identity are very different in receptor dependence, with vB_VpaM_R20L utilizing flagellar motility for adsorption to the receptor and vB_VpaM_R19R accessing the receptor through a static LPS epitope [89]. This functional divergence shows that very small differences in the phage’s sequences can result in a change in the direction of the selective pressure against the host, affecting its receptor system. See Table 2.

**Figure 3:**
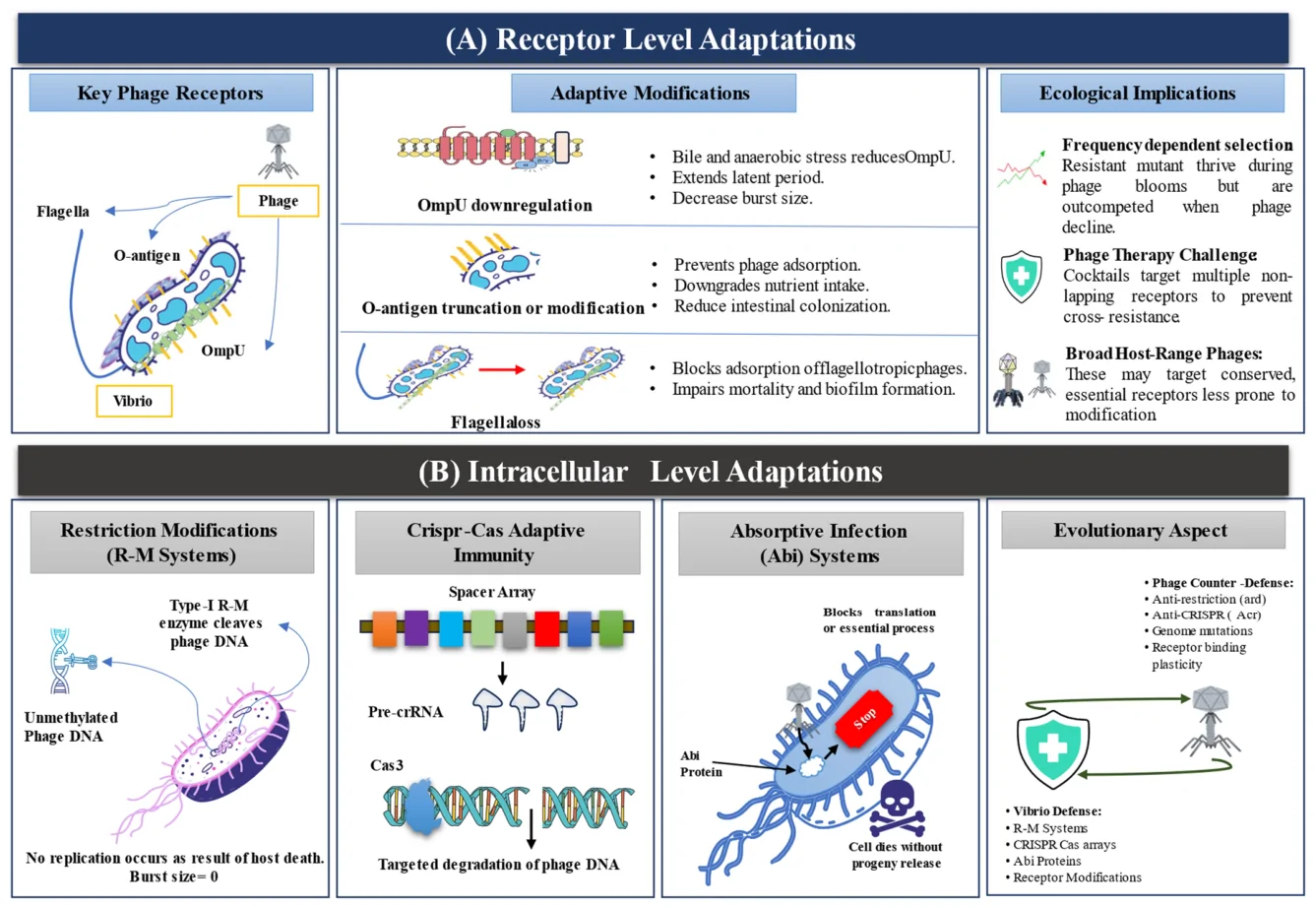
Adaptive modifications (**A**) Receptor Level Adaptations and (**B**) Intracellular Level Adaptations.

There are far-reaching ecological consequences for receptor-level adaptations. These changes tend to improve the fitness of the bacteria in the absence of phage,] and thus result in frequency-dependent selection; when the number of phages is high, resistant mutants will be more fit and will outcompete susceptible mutants when phage is scarce. This coevolution is cyclical and helps maintain the genetic diversity of *Vibrio* populations and regulate the height and length of lysis events in natural systems. Furthermore, in a therapeutic point of view, the fact of receptor plasticity constitutes a major problem for the design of phage therapy cocktails, since the development of mutants resistant to one phage will be less likely to be resistant to the other ones. The recent characterization of phage SSJ01, which rapidly binds to *V. parahaemolyticus* over a wide range of salinity and pH, suggests that it may target a highly conserved and essential surface structure that is less likely to be modified and provide a template to generate more robust therapeutic phages [91]. Figure 3 shows the adaptive modifications at receptor and intracellular levels.

To conclude, receptor-level adaptation in the interaction between *Vibrio* and phages is not just a simple on/off mechanism of infection but an environmentally sensitive adaptation, that is, a trait that is dynamic in relation to the survival of the organism and its ecological competitiveness. The effect of their action on the efficiency of lysis is straightforward and measurable: the less they are adsorbed, the smaller proportion of cells will enter the lytic cycle, and hence the burst output of the phage will decrease in a linear fashion and the temporal kinetics of phage propagation will be changed. These mechanisms are crucial for understanding the dynamics between phages and bacteria in natural environments and when applied to aquaculture disease management.

### 4.2. Intracellular Defense Systems

Second, intracellular defense systems, such as restriction-modification (R-M) systems, CRISPR-Cas arrays, and abortive infection (Abi) modules, block phage replication after the phage is adsorbed. When *V. alginolyticus* is subjected to phage selection, it quickly activates Type I R-M systems that cleave off the incoming phage DNA before any duplication has occurred, thus reducing the burst size to zero in a subpopulation of infected cells within the culture [73].

The simplest form of intracellular defense is the restriction-modification system. In *V. alginolyticus*, exposure to lytic phages quickly selects for strains with up-regulated Type I R-M enzymes that recognize and cut unmethylated phage DNA in minutes after the phage is injected into the cell. This enzymatic degradation prevents replication before early gene expression, leading to the complete blockage of holin-endolysin synthesis and, therefore, to zero production of progeny. More importantly, this process is often accompanied by the death of the host cell through the activation of toxins, making the infected cell a “dead end” for the phage, which reduces the burst size to zero in infected subpopulations. Transcriptomic profiling indicates that the nucleotide biosynthesis pathways are also concurrently down-regulated, possibly indicating metabolic reallocation towards defense rather than support for replication [73].

Abortive infection (Abi) mechanisms are “scorched-earth” mechanisms in which the infected cell “dies” before it can infect others with the virus. In *Vibrio*, there are emerging indications of similar pathways, while in Lactococcus and Bacillus, they are better described. Some *V. parahaemolyticus* strains, for example, contain similar proteins that control the expression of the *E. coli* lit system, which prevents elongation on arrival of the phage T4 transcription factor analog [94]. Although there is limited direct evidence for this in *Vibrio*, assays of single-cell lysis timeline show mixed results, with some cells undergoing rapid lysis (about 15 min post-infection) and others dying without releasing virions, consistent with Abi activation 4. This phenotypic diversity leads to a coefficient of variation in lysis time of <0.1 for susceptible strains and >0.3 for resistant strains, which compromises the synchrony needed for explosive propagation of phages [95].

These intracellular defenses come at major fitness costs. In nutrient-limited conditions, R-M upregulation consumes ATP and S-adenosyl methionine for methylation reactions that do not support growth, leading to an 18–25% decrease in doubling rates. CRISPR maintenance imposes an energetic cost because of the continuous expression of the cas genes and the possibility of genome instability caused by the acquisition of spacers. In outbreaks, designed to kill a portion of the population, Abi systems reduce carrying capacity. However, under variable conditions of phage pressure, these costs are balanced by increased survival in epidemics [16].

To conclude, *Vibrio*-phage intracellular defense systems do not only prevent the cell from being lysed, but they also alter the fate of the cell after infection, thereby inducing abortive, delayed, or heterogeneous outcomes. They have a large effect on lysis efficiency: they decrease the fraction of cells to which the virus can attach and infect, which decreases effective burst size, and also desynchronize the timing of lysis, which slows the rate of phage population growth. This “immune checkpoint” of the intracellular environment is important to understand when designing effective phage therapeutics since cocktails need to deal with not only the adsorption barrier but also this “immune checkpoint” to ensure a reliable clearance of the pathogen.

### 4.3. Mutations in Core Lysis Genes

Thirdly, mutations in core lysis genes or their regulation result in “lysis-defective” phenotypes that uncouple replication from release. Mutations in lysis genes result in the ability to infect the host cell through genome replication and virion assembly, but the timely and exact execution of host cell rupture is disrupted. This “late-stage sabotage” will preserve the cellular integrity for long enough to allow limited spread of phages and may allow alternate means of survival beyond simple lysis, such as lysogeny or abortive infection. These mutations have been found, in recent studies on several *Vibrio* pathogens, to primarily affect three proteins of the canonical lysis cassette, which regulate different biophysical events of envelope disintegration: holin, endolysin, and spanin proteins.

Less frequently, endolysin mutations also help for lysis evasion because of their enzymatic essentiality. After holin-mediated inner membrane permeabilization, endolysins are capable of breaking bonds in peptidoglycan. Transcriptomic comparison of the expression profiles of *V. alginolyticus* phage-resistant strains shows that the genomes of these strains contain less expression of host-encoded amidases that are frequently used in, or complemented by, the activity of phages during lytic infection. Mutations in host-encoded cell wall-degrading enzymes are rare in host genomes (because they are viral genes), while mutations in the genes for peptidoglycan cross-linking (or peptidoglycan acetylation) may make them insensitive to endolysin activity [96]. This indirect resistance to endolysin function is “phenotypic resistance” because it is not a consequence of genetic changes in the lysis cassette itself but rather due to structural fortification.

The spanin mutations are a new frontier in lysis interference, especially for outer membrane Gram-negative *Vibrio*. After endolysin cleavage, the disruption of the outer membrane is completed by spanins, which are either unimolecular (u-spanins) or two-component (i-spanins and o-spanins) [88].

These lysis gene adaptations come with large fitness costs. Delayed lysis causes a prolonged metabolic load of maintaining phage replication, which results in fewer resources available for growth and stress responses. Additionally, mutations in holin or spanin genes could have pleiotropic effects on the native membrane transport or envelope integrity and make cells more vulnerable to osmotic shock or antibiotics. In environments where phage pressure varies over time, however, these costs are more than compensated for by increased survival during epidemics, as in estuarine ecosystems or in the human gut. Of critical importance is that lysis gene mutations occur downstream of the DNA injection step and may not hinder horizontal acquisition of virulence or resistance gene components by transduction in “resistant” populations. The coevolutionary view is that these adaptations are selecting for phages with modified lysis cassettes, such as holins with modified triggering thresholds or anti-spanin suppressors, thus driving an arms race that diversifies host and viral genomes [92]. This is important for the design of phage therapeutics, which can avoid the dependency on lysis (such as by the use of phage-encoded depolymerases) or integrate more than one lysis mechanism in order to ensure that the host is unable to sabotage its own destruction. Finally, mutations in core lysis genes are examples of how the infection can be separated from destruction: mutations that allow the bacteria to escape a lethal interaction into a controlled and survivable event.

### 4.4. Physiological Adaptations

Fourth, physiological adaptations alter metabolic fluxes, which inhibit phage development. Lysis efficiency is closely linked to carbon and nitrogen metabolism: many enzymes encoded by phages exploit intermediates of the TCA cycle and amino acid biosynthesis pathways for nucleotide and capsid protein biosynthesis. The 40% decrease in the burst size for CCR mutants in *V. cholerae* during infection under glucose-limited conditions results from the decreased glycolytic flux, which in turn decreases the supply of ATP needed for genome packing [97]. In contrast, a greater number of phages encoded nitrogen assimilation genes were activated in the presence of nitrogen-rich conditions, raising the burst size by 2.3-fold, which illustrates that lysis efficiency is not an intrinsic property of the phages but rather is an emergent property of the phage–host–environment triad [98].

*Vibrio* phage lysis is not genetically programmed to time and place to occur but rather is dynamically regulated in response to the physiological status of the host bacterium and thus reflects a high level of environmental sensing and response. Many *Vibrio* phages, such as JSF4 and VP882, postpone lysis in nutrient-poor environments (typical open ocean habitats) by either expressing anti-holin proteins or by using small RNA (sRNA) to inhibit translation of holin. This physiological delay synchronizes the size of the burst with the capacity of the host ribosomes and other biosynthetic resources to produce the maximum number of viable progenies before the host’s metabolic ability is depleted. This strategy comes directly from the “resource limitation hypothesis” which suggests that lysis in a starved host will produce a small, non-competitive burst. In addition, quorum-sensing cross-talk is a mechanism for population-level control. Phage VP882 encodes the host’s CAI-1 autoinducer molecule which is a major quorum-sensing signal molecule, in *Vibrio* spp. The phage contains Qrr sRNAs, which are activated by CAI-1 and which repress the holin gene [89]. Therefore, lysis will be inhibited at low cell densities (low CAI-1) and only occur at high cell densities, where there will be more virions available for immediate encounter with new hosts. The physiological influence of osmotic adaptation is also of major importance. The phages of Marine *Vibrio* hosts have evolved lysis proteins that are stable and active in high-salinity water. The endolysins are also halostable, such as the ones with more surface acidic residues but a less hydrophobic core, making them more soluble and avoiding aggregation in salt solutions. The adaptation is vital in aquaculture, where salinity levels can differ greatly, and it cannot be adapted, or it will not be able to lyse the bacteria in the aquaculture environment [99].

To sum up, the physiological adaptation of a *Vibrio*–phage system acts as a dynamically modifiable “metabolic firewall”, operating by environmental sensing and resource allocation to control the efficiency of lysis. *Vibrio* cells can transiently switch to states that are unproductive for infection without making permanent genetic changes, allowing switching between immediate survival and long-term ecological competitiveness while incorporating signals based on nutrient status, stress, and host niche. The knowledge of these mechanisms is crucial for understanding and predicting the outcomes of phage therapy in complex habitats such as the gut or aquaculture pond, where not only the host genotype (genus/species) but also its specific physiological conditions define the effectiveness of the therapy.

### 4.5. Structural Adaptations

Fifth, structural changes modify the biophysics of membranes to make it more difficult for holins to function. The thermos-responsive stiffness changes that occur in phage C22 are not specific to *Vibrio* but offer a mechanism: *Vibrio* membranes containing more unsaturated fatty acids become more fluid in the cold, favoring holin oligomerization and early lysis, and more rigid in the heat, delaying pore formation. *V. alginolyticus* stress response activation through RpoS has resulted in higher production of cardiolipin, which has been shown to harden regions of the membrane and decrease the diffusion coefficient of holins by 37%, thus prolonging the latent period and decreasing the burst fidelity [73].

These adaptations work together to influence the life history characteristics of viruses. Sustained phage pressure results in *Vibrio* populations evolving from high-burst, short-latent-period to low-yield, temporally dispersed lysis, as seen in phage–host coevolution experiments, which leads to lower peaks of phage titer but increases persistence in a fluctuating environment. Structural changes that occur during phage-*Vibrio* interactions are an important mechanism of host defense, but one that is often overlooked, as it is mediated by physical and biophysical modification of the *Vibrio* cell envelope that directly interferes with the final steps of the phage lytic cycle. Structural adaptations change the biophysical characteristics of cell membranes and cell walls (e.g., by disrupting the necessary spatiotemporal coordination of holin pore formation, access to endolysin, and outer membrane rupture), rather than by blocking phage adsorption or DNA injection, as occurs with genetic or receptor-level resistance mechanisms [100]. In Gram-negative *Vibrio* species like *V. alginolyticus*, *V. cholerae,* and *V. parahaemolyticus*, the double membrane structure of their cell walls requires a complex three-part lysis system (holin–endolysin–spanin) to provide complete cell disruption.

To conclude, the structural changes in *Vibrio*–phage systems could be considered a biophysical “armor”, which dynamically regulates the lysis efficiency and thus the physical environment in which holins, endolysins, and spanins act. This coupling of environmental stress signals into the membrane and wall architecture allows *Vibrio* cells to temporarily protect against complete lysis (without permanent genetic alteration) and thus provide a compromise between immediate survival and long-term ecological competitiveness. In more complex settings where the therapeutic efficacy is determined not only by the host genotype but also by the host physiology, it is crucial to understand these mechanisms to predict the outcome of phage therapy.

## 5. Challenges

**Figure 4:**
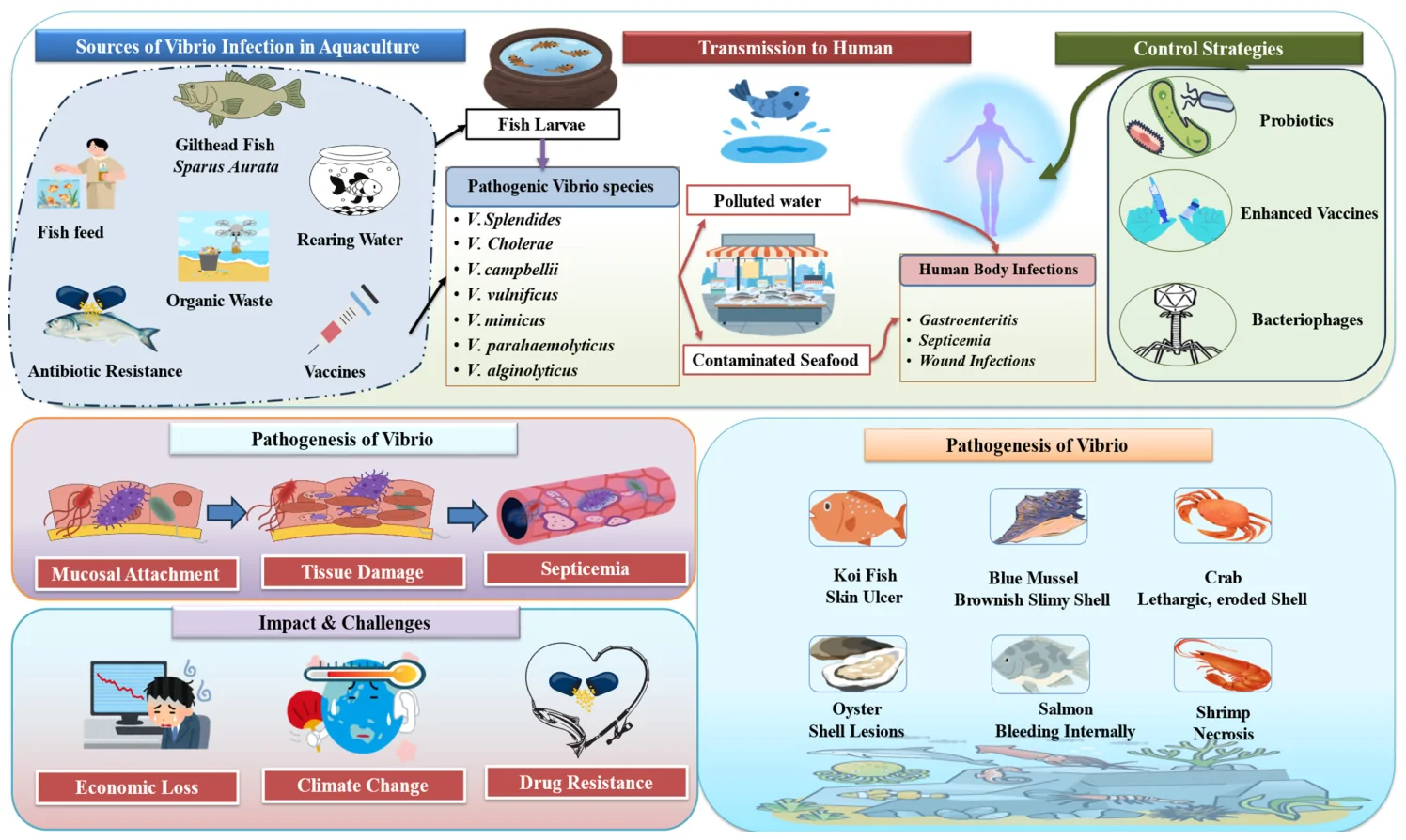
Sources, Transmission, and Pathogenesis of *Vibrio* in Human, and Aquaculture.

The population of *V. crassostreae*, a species associated specifically with oysters, was genetically structured into clades of near-clonal strains, leading to the isolation of closely related phages forming large modules in phage-bacterial infection networks. Small modules in the phage-bacterial infection network were found for *V. chagasii*, which flourishes in the water column, due to the low number of closely related hosts and high diversity of isolated phages. Phage load was correlated with *V. chagasii* abundance over time, suggesting that host blooms may influence phage abundance. Genetic experiments also showed that such blooms of phages can produce epigenetic and genetic changes that can lead to resistance against host immune systems [101] The outcomes underscore the significance of taking into account the environmental dynamics and also the microbial community structure of the host while analyzing phage-bacteria networks. In this study, it was explored how different genetic structures of hosts can feed back on host-phage interaction networks and genetic diversity of both hosts and phages. The genetic diversity of both hosts and phages, in different conditions of different host species, led to different structures of the respective phage-bacteria interaction networks. See Figure 4.

Bacteriophages are gaining significant interest as an alternative to antibiotics in aquaculture, seafood processing, and environmental biotechnology for the treatment of pathogenic *Vibrio* species as a sustainable solution. However, despite significant progress in the understanding of phage adsorption, specificity of the phages, lytic machinery, and host adaptation, there are a number of fundamental obstacles that prevent the transfer of *Vibrio*-phage systems from the lab to stable commercial systems. Some of the main problems are the absence of standardized lists of phage resistance characteristics, insufficient knowledge of phage marine ecology, and the absence of harmonized regulatory systems for the production and environmental use of phages [102]. Such constraints play a significant role in terms of the efficiency of lysis, prediction of the susceptibility of hosts, the ecological stability of a system, and the reproducibility of the therapeutic action of phages in laboratory-scale studies or industrial-scale applications.

A major scientific hurdle in the study of *Vibrio*-phage is the lack of a universally standardized database that has been assembled that could combine *Vibrio* receptor variability, adsorption kinetics, host range specificity, and lytic activity. Phage resistance characterization is currently far from consolidated between different laboratories, unlike the case of the antibiotic resistance systems, for which curated international repositories exist and standardized breakpoints for resistance are used [103]. Such a limitation is especially problematic for *Vibrio* species, which are genetically very flexible due to horizontal gene transfer, mobility, recombination, and adaptation to their environment. Consequently, a study of the susceptibility profiles of several *Vibrio* strains reveals significant variations within and between species, including *V. cholerae*, *V. parahaemolyticus,* and *V. alginolyticus*, as well as between closely related strains from different aquaculture systems or geographic areas.

An interesting example in *V. parahaemolyticus* is the repeated exposure of a mixed population of phages that resulted in the selection of a strain with mutations in capsular polysaccharide biosynthesis genes, such as ugd, gtr, and gtr2. These mutations resulted in changes in the cap’s structure, which decreased phage adsorption and led to the loss of lytic efficiency. The same mutations also increased biofilm production, and the changes in colony morphology, however, indicated that resistance mechanisms did not simply involve the loss of receptors but rather complex physiological trade-offs. Importantly, the data on resistance from such studies are not included in a unified database with standardized phenotypic and genomic descriptors, making it difficult to design effective phage cocktails [104]. Commercial formulations based on strains from one part of the world may thus not be suitable for genetically distinct strains of *Vibrio* found in other parts of the world. The lack of standardization also makes it difficult to reproduce the results performed in the lab, because the results of phage susceptibility measurements often depend on the multiplicity of infection (MOI), the salinity, the temperature, the growth phase, and the composition of the media, making it difficult to compare the results from different studies.

Another important challenge is the complexity of marine phage ecology, which hinders scalability and predictability of *Vibrio*-phage lysis systems. Marine ecosystems are one of the most dynamic and constantly changing microbial communities on Earth, which are further influenced by salinity, temperature, nutrient availability, seasonal changes, wavelengths, and microbial competition between host and phage [105]. These environmental factors have a significant effect on phage adsorption kinetics, latent period, burst size, and lytic efficiency. This means that phages with high lytic activity in laboratory settings often do not work as effectively in aquaculture settings.

Longitudinal sampling studies in marine aquaculture systems have shown an important ecological example of the populations of oyster-associated *V. crassostreae* and free-living *V. chagasii*. The results showed that host populations with a higher genetic diversity and more environmental diversity of *Vibrio* bacteria harbored wider but less stable phage infectivity networks, while structured populations of hosts generated very modular phage-bacteria interaction networks. In addition, the abundance of the phages closely paralleled the dynamics of the bacterial blooms, indicating that phage growth and adaptation to their environment are strongly influenced by environmental fluctuations [106]. The results showed that lytic activity in natural environments is more complex than can be determined by laboratory plaque assay, and natural ecosystems impose ecological constraints that are not met in simplified in vitro systems. This ecological variation creates significant challenges for commercial aquaculture applications because variations in temperature or salinity from season to season could significantly affect the efficacy of the phages and lead to the evolution of phage resistance.

In contrast, low-cell-density variants were found to be more susceptible to lysis and to have a higher level of expression of OmpK. The interesting point is that the resistant populations responded by increasing their biofilm production and not by a permanent genetic change, so that nonmutational resistance strategy could be more important in natural ecological conditions [107]. This receptor accessibility regulation is density-dependent, leading to significant variability in phage lysis efficiency in commercial aquaculture operations, where the bacterial population density is constantly changing.

**Figure 5:**
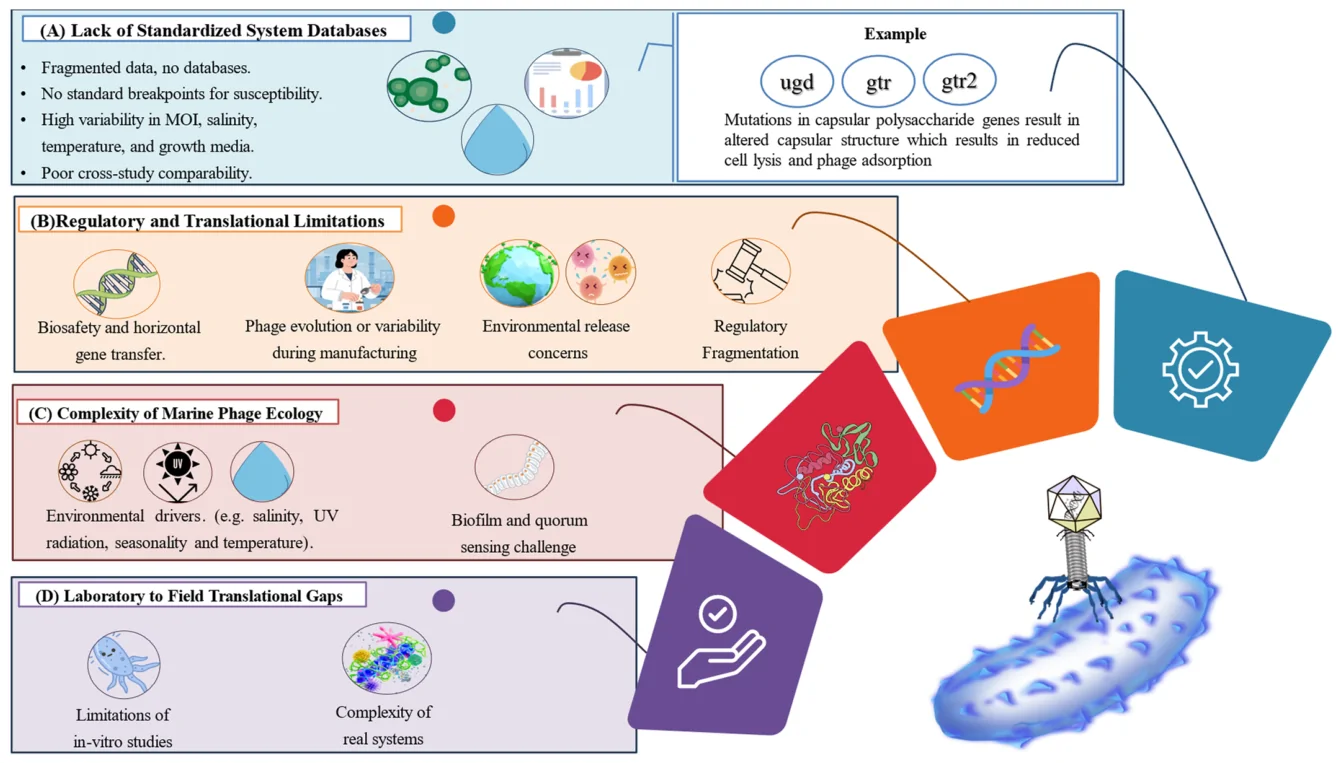
Challenges; (**A**) Lack of Standardized System Databases, (**B**) Regulatory and Translational Limitations, (**C**) Complexity of Marine Phage Ecology, and (**D**) Laboratory to Field Translational Gaps.

The main concerns with regulation are biosafety and horizontal gene transfer. Temperate phages can integrate into the bacterial genome and could transduce virulence determinants, toxin genes, or antimicrobial resistance genes. This is especially true for *V. cholerae*, which exhibits a high correlation between pathogenicity and lysogenic phages with cholera toxin genes, like CTXϕ [108]. Regulatory agencies thus strongly prefer only strictly lytic phages to be used as therapeutics, but the distinction between strictly lytic phages and cryptic temperate variants is only possible to make through a thorough genomic and functional characterization. This adds to the costs of development and slows down the commercialization process. Figure 5 shows different challenges related to phage *Vibrio* implications and systems.

Regulatory challenges are significant with manufacturing consistency as well. For commercial production of phage, the prerequisite conditions are the following: the host strains used must be stable, propagation conditions must be reproducible, endotoxin removal is required, and there must be an assurance of genetic integrity between production batches. In natural propagation, though, phages evolve naturally, especially at high densities during fermentation. Lytic properties may be changed in industrial production due to mutations in tail fibers, recombination of the receptor-binding proteins, or spontaneous host-range expansion [109]. During initial development, a customized phage cocktail might have an optimized infectivity profile; this may be altered after large-scale manufacturing, thus making quality control and regulatory validation difficult.

Further complicating regulatory approval is the issue of environmental release. The use of very high levels of phages in aquaculture systems can raise concerns about the potential for unintended impacts on marine ecosystems. The potential for microbial community structure, nutrient cycling, and non-target bacterial populations to be altered by the dissemination of phages at larger scales is not well understood [110]. Thus, detailed environmental impact evaluations are needed by the regulatory agencies, but doing so is challenging due to the fact that marine phage ecology itself is still poorly understood. The observed impact of the lytic phages is to decrease the susceptible population of the *Vibrio*s through infection and cell lysis. Biofilms and pathogenicity associated with *Vibrio* can be decreased in experimental aquaculture systems by phage treatment. In some cases, phage treatment can change the composition of gut or environment bacteria, and the direction can vary. This is a broad process known as phage-mediated (intracellular) lysis that is widely known in the ecology of marine viruses. Conversely, the potential ecological effects are possible consequences that have not been well established for widespread therapeutic *Vibrio* phage use. These include long-term consequences of disseminating phages at the ecosystem level, significant alteration of beneficial/non-target bacteria, disruption of carbon, nitrogen or phosphorus cycling and persistent alteration of the overall microbial community [9]. The two *V. parahaemolyticus* phages were able to disrupt the biofilms of eight V. non-host species as well as multispecies *Vibrio* communities without being able to productively infect these microorganisms [29]. To sum up, a large-scale application of *Vibrio* phage could be a possible influence on the structure of microbial communities and nutrient cycling, but this effect has not been proven yet to occur in the ecosystem level of the *Vibrio* phage application in therapy.

There are also significant methodological constraints in lab-based research that need to be addressed for successful field-scale implementation. Numerous in vitro studies have been conducted with single species grown under strictly controlled conditions, which do not represent the complexity of the marine environments. Microbes are highly sensitive to the characteristics of the environment, including salinity, nutrient availability, microbial competition, biofilm formation, and host physiological state, which significantly affect lysis dynamics but are poorly captured in laboratory experiments [111]. Phages with good plaque-forming ability in the laboratory then do not necessarily show the same ability in an actual aquaculture setting. This difference shows the importance of establishing integrated experimental systems where environmental simulation, metagenomics, transcriptomics, and systems biology approaches are combined to provide more accurate models of natural interactions of *Vibrio* and their phages.

All these challenges show the need for a multidisciplinary approach that combines molecular microbiology, marine ecology, genomics, systems biology, bioinformatics, and regulatory science to realize the implementation of *Vibrio*-targeting phages. The development of global databases of phage resistance, together with ecological surveillance networks and established harmonized regulations, will play a key role in enhancing the reproducibility and commercial reliability. Additionally, new technologies like artificial intelligence-based host prediction, synthetic phage engineering, cryo-electron microscopy, and multi-omics profiling could overcome several of the current constraints by allowing precision phage therapy in specific environmental and pathogenic conditions. In the end, phages need to overcome these intertwined scientific and regulatory challenges to make the field of phage therapy for *Vibrio* species a viable and sustainable commercial technology in aquaculture and food safety.

## 6. Future Perspective

**Figure 6:**
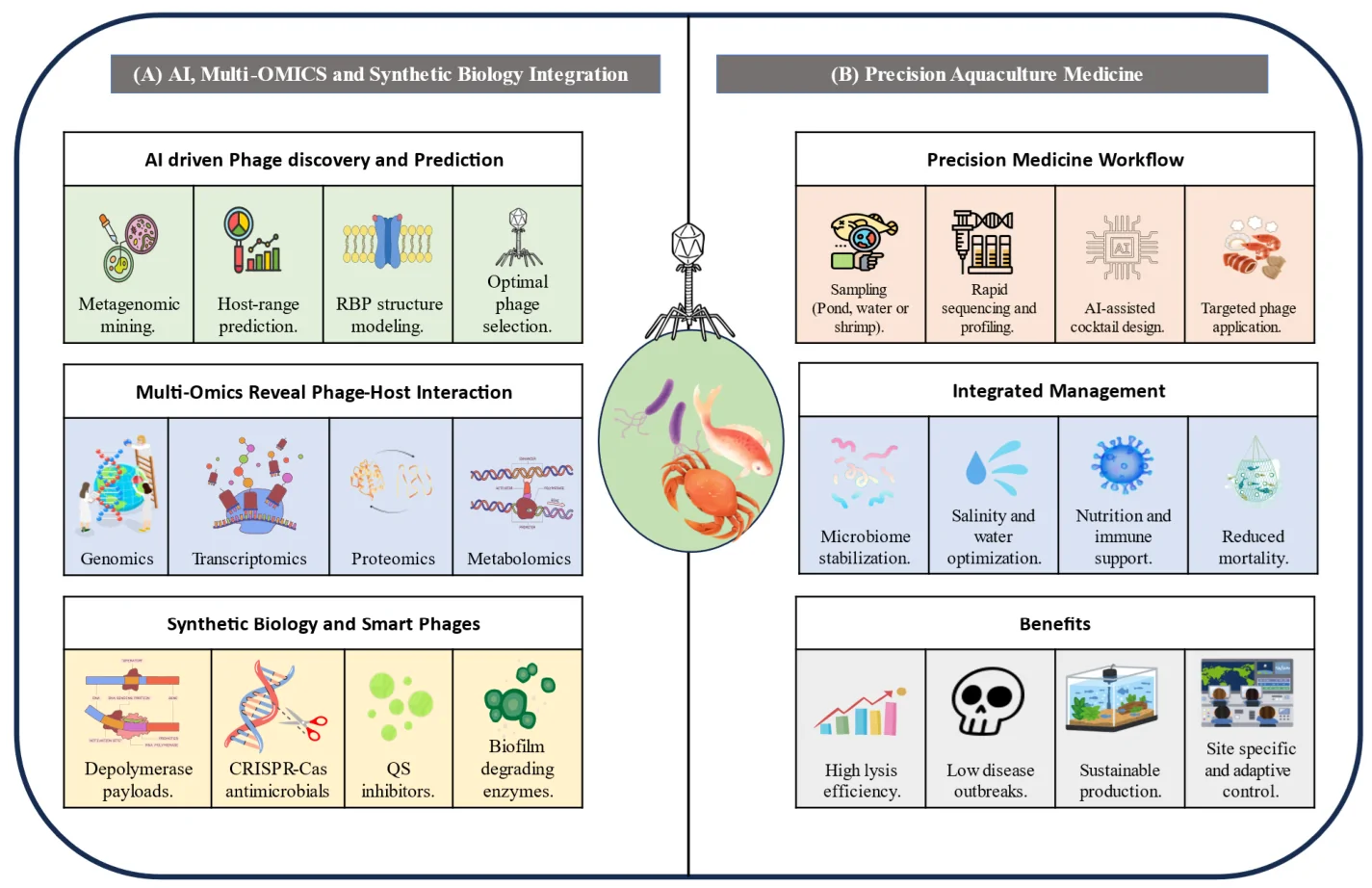
The future of *Vibrio*-phage control systems; (**A**) AI, Multi-OMICS, and Synthetic Biology Integration, and (**B**) Precision Aquaculture Medicine.

The success of the bacteriophage-based control strategies against pathogenic *Vibrio* spp. will rely not only on the enhancement of lytic activity but also on the evolutionarily stable and ecologically adaptable and technologically integrated therapeutic systems. While the promise of phage therapy as an alternative to antibiotics in aquaculture and food safety is promising, several emerging challenges, including phage evolution, host adaptation, environmental variability, and therapeutic precision, must be addressed in order to achieve long-term sustainability. Within this context, 3 major future directions are predicted to dominate the future research on *Vibrio*-phages: the evolutionary stability of engineered *Vibrio*-phages, the evolution of ‘precision aquaculture medicine’, and the incorporation of artificial intelligence (AI), multi-omics technologies, and synthetic biology in phage discovery and therapeutic design [112]. Figure 6 shows the future of *Vibrio*-phage control systems.

An evolutionary stability of engineered phages is one of the most crucial future factors. The rates of evolution of natural bacteriophages are high, partly because of the rapid evolution of their receptors with the rise of bacterial resistance and partly because of the emergence of other resistance mechanisms, including CRISPR-Cas immunity, restriction-modification systems, quorum sensing-regulated defense pathways, and biofilm formation. Although synthetic biology has now made it possible to engineer receptor-binding proteins (RBPs), lysis modules, host-range determinants, and anti-defense systems, a clear challenge is how to maintain the stability of these engineered features for long-term applications [113]. In marine environments, *Vibrio* can be genetically very promiscuous and acquire new genes through horizontal gene transfer, which can lead to the rapid loss of efficacy of engineered phages.

One such case study is the evolutionary arms race between *V. cholerae* and the ICP1 phage. ICP1 has its own CRISPR-Cas system to specifically target phage-inducible chromosomal island-like elements (PLEs) in *V. cholerae*, which evades the bacterial antiviral defense system. *V. cholerae*, however, constantly generates new PLE variants that escape recognition by the CRISPR, requiring “reciprocal adaptation” in the phage [92]. The ability of such highly specialized phages equipped with sophisticated anti-defense systems to be rapidly adapted by their host suggests that coevolution is a dynamic process that can be unstable and easily outpaced. These discoveries resonate with the idea that static phage-based designs will likely become obsolete in commercial aquaculture environments where bacteria are highly dynamic and subjected to strong environmental and therapeutic stresses.

Effective countermeasures available to date in aquaculture are carried out in a broad sense with general-purpose antibiotics or probiotics, which is based on applying the same treatment for all populations irrespective of pathogen genotype, environment, and host immune status. Precision aquaculture medicine, on the other hand, aims to customize interventions based on the particular microbial ecology, pathogen diversity, host physiology, and environmental conditions of individual aquaculture systems [114]. The high host specificity and adaptability of phages make them particularly suitable for such an approach.

Precision aquaculture medicine thus imagines the development of real-time monitoring of microbial populations based on environmental sequencing, metagenomics, and biosensor technologies. For instance, in the aquaculture industry, dominant *Vibrio* strains and their resistance determinants could be rapidly identified right in the facility using portable nanopore sequencing systems. Computer screening of phage libraries could then be used to find the best lytic combinations to fight with locally circulating pathogens [115]. These will greatly enhance lysis efficiency and reduce emergence of resistance.

A good example of this is the application of *V. parahaemolyticus* phage cocktails in shrimp farms to control acute hepatopancreatic necrosis disease (AHPND). It was found that phage formulations designed to be specific to strains of the outbreak in a particular region were much more effective at saving lives than commercial cocktails designed to be effective against a broad range of known strains. In addition, the integration of phage therapy with environmental management measures like salinity optimization and microbiome stabilization contributed to improved long-term pathogen management [116]. The results indicate that future aquaculture systems could be configured like precision medicine in human medicine, where therapeutic treatments are continually adapted in response to changing pathogen characteristics and feedback from the environment.

Receptor-binding proteins are now being analyzed using machine learning algorithms, and the specificity of their adsorption is predicted, and candidate phages are being pulled from metagenomic databases. Even though very few *Vibrio* species can be cultured in the laboratory, there are vast amounts of uncultured sequences in marine environments, from which AI-based host predictions can be made without needing to culture them. Deep learning platforms based on genomic and structural information are also under development to predict interactions between the receptors and LPS, capsular polysaccharides, pili, and outer membrane proteins (OmpU and OmpK) [73]. These technologies could make the selection of effective phages in aquaculture outbreaks much faster.

Enabling the design of “smart phages” with programmable functionalities further expands these possibilities, thanks to synthetic biology. Future engineered phages could contain quorum-sensing inhibitors, metabolic regulators to make bacteria more susceptible to lysis, enzymes to degrade biofilms, and CRISPR-based antimicrobials. Multifunctional phages could be used in aquaculture to lower pathogen numbers, disrupt pathogen-associated biofilms, inhibit expression of virulence genes, and change the composition of the microbial community [113]. An interesting example of such an approach is the engineering of depolymerase-producing phages against capsular polysaccharides of *V. parahaemolyticus*. These phages destroy the protective biofilm and capsule structures during adsorption, with a greater efficiency for penetration and lysis in dense microbial populations [21]. There are also other CRISPR-Cas payload-based strategies that have been shown to selectively kill antibiotic resistance plasmids in Gram-negative pathogens, which could have future applications in controlling multidrug-resistant *Vibrio* strains.

These integrated technologies have the potential to transform aquaculture biosecurity systems in a commercial setting. In the future, such automated monitoring platforms integrating environmental sensors, AI-driven outbreak prediction, metagenomic surveillance, and adaptive phage deployment could deliver real-time disease management in intensive farming [117]. Laboratory-scale research will also become more interdisciplinary, combining structural biology, computational modeling, systems microbiology, and synthetic genomics, to create very efficient and optimized phage therapeutics. In conclusion, the prospect of the future of *Vibrio*-phage lysis research is to go beyond classic phage therapy and towards eco-engineered interventions tailored to the individual. Together, these evolutionary stable phages, customized aquaculture treatments, and AI-powered synthetic biology tools are the future of sustainable disease management [118]. Such strategies are not only more efficient lysis methods and decrease antibiotic reliance but can also fundamentally change the ecological and technological landscape of modern aquaculture and marine biotechnology.

## 7. Conclusions

This review highlights that the success of phage-mediated lysis is strongly dependent on the accessibility and diversity of bacterial structural receptors, including receptor-binding proteins (RBPs), lipopolysaccharides (LPS), capsular polysaccharides (CPS), porins, flagella, pili, and outer membrane vesicles (OMVs). These receptors not only determine phage adsorption efficiency and host specificity but also influence the evolutionary dynamics between phages and their bacterial hosts. Simultaneously, *Vibrio* species possess highly adaptive defense strategies such as receptor modification, quorum-sensing regulation, biofilm formation, intracellular anti-phage systems, and mutations in lysis-associated genes, all of which contribute to resistance development and altered lysis efficiency.

Recent advances in genomics, transcriptomics, whole-genome sequencing, adsorption kinetics assays, and AI-assisted computational tools have significantly improved understanding of phage-host interactions and resistance mechanisms. However, important challenges remain, including limited knowledge of marine phage ecology, insufficient standardized resistance databases, and regulatory limitations for therapeutic applications in aquaculture systems.

Overall, integrating engineered phages, synthetic biology, multi-omics technologies, and artificial intelligence with precision aquaculture medicine may provide a new generation of sustainable and evolutionarily stable solutions for controlling *Vibrio* infections. A deeper understanding of receptor accessibility, host adaptations, and phage evolutionary responses will be essential for improving lysis efficiency, minimizing resistance emergence, and advancing the practical implementation of phage therapy in modern aquaculture and food safety management.

## Figures and Tables

**Table 1:** Summary of experimentally characterized *Vibrio* phage receptors and resistance mechanisms involved.

Sr. No.	*Vibrio* Species or Strains	Type of Phage	Host Receptor	RBPs	Resistance Mechanism	Evidence Supporting Receptor Identification	References
1.	*V parahaemolyticus*	vB_vpM-PP270	CPS	ORF22 tail spikes protein	CPS synthesis gene mutation changes capsule structure and decreases the phage adsorption.	CPS deficient mutants showed decrease in adsorption resistance. Mutation in ugd, gtr and gtr2 initiated the resistance. ORF22 prevented the adsorption and directly interacted with host’s CPS.	[21]
2.	*V. alginolyticus* E110	HH109	CPS	gp02; tail protein	Functional mutations in CPS genes including wecA and orf2, reduce or eliminate capsule production leading to phage adsorption.	Transposon screening identified CPS-associated genes; deletion of ugd, wecA, gt1, orf2 and gt6 reduced adsorption. Proteinase K treatment did not abolish adsorption, antibody-blocking and immunofluorescence assays demonstrated gp02-CPS interaction.	[22]
3.	*V. alginolyticus* VP505	φVP505	CPS	----	Reduced CPS production through mutations or deletions in CPS-associated genes, resulting in impaired adsorption.	Deletion of wecA and ugd specifically reduced adsorption, demonstrating CPS dependence. CPS-deficient mutants were resistant to infection.	[15]
4.	*V. parahaemolyticus*	OWB	Vp0980, an OMP	Tail tubular proteins A and B (TTPA/TTPB)	Mutation or deletion of vp0980, particularly alteration of its exposed outer-membrane region, prevents interaction with TTPA/TTPB and reduces adsorption.	Transposon mutagenesis identified vp0980, complementation restored phage susceptibility; adsorption and confocal assays showed reduced binding in the mutant; pulldown assays demonstrated direct interaction between Vp0980 and TTPA/TTPB).	[23]
5.	*V. parahaemolyticus*	KVP40	OmpK	----	Loss or modification of OmpK prevents phage adsorption and confers resistance.	A spontaneous KVP40-resistant mutant lacked OmpK; purified OmpK and outer membrane from the susceptible strain inactivated KVP40, whereas material from 6.the resistant mutant did not. OmpK homologues were detected across several *Vibrio* spp.	[24]
6.	*V. cholerae* O1 El Tor	VP3	LPS core oligosaccharide + TolC co-receptor	gp44 tail-fiber protein	Mutations in the TolC exposed loops and alterations/loss of LPS oligosaccharide structures reduce phage binding and infection.	Mutations in the LPS oligosaccharide region decreased adsorption and TolC mutation further reduced binding, while a double mutant abolished binding. Protein–protein interaction assays demonstrated direct interaction between TolC and gp44. Critical TolC surface residues were identified.	[25]
7.	*V. cholerae* O1 El Tor	VP1	VcpQ (PolyQ membrane protein)	----	Reduction of glutamine residues in the VcpQ PolyQ tract alters the receptor and prevents VP1 capture.	Spontaneous phage-resistant mutants and genome sequencing identified deletions in the vcpQ polyQ region; VcpQ depletion caused resistance, establishing its role as the receptor.	[26]
8.	*V. vulnificus* MO6-24/O	SSP002	Flagellum	----	Loss or disruption of flagellar assembly/function prevents receptor presentation and confers resistance.	Tn5 mutagenesis identified flhA, flhB, fliF and fleQ; deletion of these genes prevented infection, whereas complementation restored susceptibility, strongly supporting flagellum-dependent recognition.	[27]
9.	*V. alginolyticus*	BUCT549	Flagellum-associated surface structure	----	Alteration/loss of flagellar-associated structures reduces phage adsorption and infection.	Infection was associated with flagellar structures; coevolution experiments showed that phage-tolerant mutants acquired mutations affecting MSHA/flagellum-associated membrane proteins and lost flagellar structures.	[28]
10.	*V. parahaemolyticus* 108T/108R	VpT	Unidentified surface receptor	ORF44/ORF47, putative tail-fiber proteins	VpT-selected 108R became resistant through a change affecting VpT adsorption; 11.the precise genetic/molecular basis was not resolved.	VpT adsorbed to susceptible 108T but not to VpT-resistant 108R. This demonstrates that the resistance phenotype affects adsorption/receptor recognition. However, no receptor gene, receptor protein, or direct RBP–receptor interaction was experimentally identified.	[29]
11.	*V. parahaemolyticus* 108T/108R	VpR	Unidentified; distinct from the VpT-sensitive receptor	ORF17, putative tail-fiber protein	VpT resistance did not confer VpR resistance, demonstrating that the two phages use complementary infection/receptor-recognition strategies.	VpR adsorbed to both 108T and VpT-resistant 108R, whereas VpT adsorbed only to 108T. This provides functional evidence for differential receptor usage, but not definitive receptor identification.	

**Table 2:** Comparative Impact of *Vibrio* Host Adaptations and Phage-Intrinsic stability Properties on Phage Infection Dynamics and Lysis Efficiency.

Sr. No.	Approaches	Molecular Basis	Stage of Phage Cycle Affected	Effect on Adsorption	Effect on Latent Period	Effect on Burst Size	Effect on Lusis Synchrony	Net Effect on Lysis Efficiency	Remarks	References
1	Bile exposure in *V. cholerae*	Downregulation of OmpU porin	Adsorption	Significant decrease	Not determined	Not determined	Not determined	Reduced infection initiation	Limits the efficacy of oral phage therapy in intestinal environments.	[88]
2	Anaerobic Condition in *V. cholerae*	Reduced receptor availability	Adsorption	Significant decrease	Increased	Decreased	Off-Synchronized	Decrease in Lysis	Needs optimization for gastrointestinal tract implications	
3	Receptor mutation in *V. parahaemolyticus*	Mutation of vp0980 gene encoding tail tubular protein receptor	Complete loss of binding	No infection	Not determined	Not determined	Not determined	Zero Lysis	Drives rapid selection of resistant mutants.	[23]
4	Broad-spectrum susceptibility in *V. harveyi*	Presence of conserved surface receptors for phage PG288	Adsorption	High Affinity	Short (25 min)	Large (298 PFU/Cell)	Highly synchronized	High Lysis Efficiency	Promising candidate for broad-spectrum *Vibrio*sis control.	[90]
5	Biofilm formation in *V. alginolyticus*	EPS Matrix	Adsorption/Penetration	Steric hindrance reduces access	Prolonged due to diffusion limits	Reduced per cell	Asynchronous	Moderate	Requires phages with depolymerase activity.	[7]
6	Receptor mobility in *V. parahaemolyticus* at 4 °C	Membrane fluidity reduction	Adsorption	Slow Adsorption kinetics	Increased	Reduced	Asynchronous	Reduction in Efficiency	Phage SS01 is active with slow lysis.	[91]
7	Salinity variation (0–6%) in *V. parahaemolyticus*	Osmotic pressure effect on cell envelope integrity	Adsorption	Stable across range	Stable	Stable	Synchronized	High efficiency	Phage SS01 robust for aquatic environments.	
8	PH extreme (5–11) in *V. harveyi*	Stability of phage capsid and tail fibers	Adsorption	Stable at extremes	Stable at extremes	Stable at extremes	Synchronized	High efficiency over a wide pH range	Adaptable to varied aquatic and gastric	
9	Co-evolutionary resistance in *V. crassostreae*	Modular network of resistance traits	Adsorption/Replication	Variable depending on module	Variable	Variable	Variable	Dynamic equilibrium	Requires cocktail therapy to overcome modular resistance	[92]
10	*V. harveyi* (Jumbo phage)	Large genome, multiple RBPs	Adsorption	Broad host range recognition	Longer (due to genome size)	Large	Synchronous	High efficiency despite size	vB-VhaM-pir03 effective against multidrug-resistant strains	[93]

## Data Availability

No new data was created or analyzed in this study. Data sharing is not applicable to this article.

## Abbreviations

The following abbreviations are used in this manuscript:

AHPND	Acute Hepatopancreatic Necrosis Disease
Abi	Abortive Infection
AI	Artificial Intelligence
EOP	Efficiency of Plating
CPS	Capsular Polysaccharides
LPS	Lipopolysaccharides
MOI	Multiplicity Of Infection
RBPs	Receptor-Binding Proteins
R-M	Restriction-Modification
OMVs	Outer Membrane Vesicles
QS	Quorum Sensing
WGS	Whole Genome Sequencing

## References

[1] Sanches-Fernandes G.M.M., Sá-Correia I., Costa R. (2022). *Vibrio*sis Outbreaks in Aquaculture: Addressing Environmental and Public Health Concerns and Preventive Therapies Using Gilthead Seabream Farming as a Model System. Front. Microbiol..

[2] Califano G., Castanho S., Soares F., Ribeiro L., Cox C.J., Mata L., Costa R. (2017). Molecular Taxonomic Profiling of Bacterial Communities in a Gilthead Seabream (*Sparus aurata*) Hatchery. Front. Microbiol..

[3] Song X., Zang J., Yu W., Shi X., Wu Y. (2020). Occurrence and Identification of Pathogenic *Vibrio* Contaminants in Common Seafood Available in a Chinese Traditional Market in Qingdao, Shandong Province. Front. Microbiol..

[4] Huang J.F., Zeng B.Q., Liu D., Wu R.B., Zhang J., Liao B.Q., He H.L., Bian F. (2018). Classification and Structural Insight into *Vibrio*lysin-like Proteases of *Vibrio* Pathogenicity. Microb. Pathog..

[5] Trinanes J., Martinez-Urtaza J. (2021). Future Scenarios of Risk of *Vibrio* Infections in a Warming Planet: A Global Mapping Study. Lancet Planet. Health.

[6] Borges N., Keller-Costa T., Sanches-Fernandes G.M.M., Louvado A., Gomes N.C.M., Costa R. (2021). Bacteriome Structure, Function, and Probiotics in Fish Larviculture: The Good, the Bad, and the Gaps. Annu. Rev. Anim. Biosci..

[7] Tadeu A.D., Duarte J., Trindade D., Costa P., Venâncio C., Lopes I., Oliveira V., Gomes N.C.M., Almeida A., Pereira C. (2024). Bacteriophages to Control *Vibrio* Alginolyticus in Live Feeds Prior to Their Administration in Larviculture. J. Appl. Microbiol..

[8] Chen Y., Li W., Shi K., Fang Z., Yang Y., Zhang R. (2023). Isolation and Characterization of a Novel Phage Belonging to a New Genus against *Vibrio* Parahaemolyticus. Virol. J..

[9] Ding H., Shi K., Hsiao M., Li W., Liu X., Xu J., Yang Y., Zhang R. (2025). Two Virulent *Vibrio* Campbellii Phages with Potential for Phage Therapy in Aquaculture. BMC Microbiol..

[10] Li H., Zhong W., Zhang X., Rui Z., Yang Y., Xu J., Gao J., Zhou X., Wu J., Xu J. (2024). Isolation and Characterization of a Novel *Vibrio* Phage VB_ValA_R15Z. Curr. Microbiol..

[11] Romero J., Blas-Chumacero S., Urzúa V., Villasante A., Opazo R., Gajardo F., Miranda C.D., Rojas R., Romero J., Blas-Chumacero S. (2024). Lysin and Lytic Phages Reduce *Vibrio* Counts in Live Feed and Fish Larvae. Microorganisms.

[12] Phan T., Shrestha A., Schow J., Miller C.R., Peters T.L., Van Leuven J.T. (2026). Bacteriophage Density Influences the Rate of Resistance Evolution. Bull. Math. Biol..

[13] Cevallos-Urena A., Kim J.Y., Kim B.S. (2023). *Vibrio*-Infecting Bacteriophages and Their Potential to Control Biofilm. Food Sci. Biotechnol..

[14] Mathieu-Denoncourt A., Duperthuy M. (2025). Functional Versatility of *Vibrio* Cholerae Outer Membrane Proteins. Appl. Microbiol..

[15] Li X., Zhang C., Li S., Liang S., Xu X., Zhao Z. (2024). Quorum Sensing Positively Regulates CPS-Dependent Autographiviridae Phage Infection in *Vibrio* Alginolyticus. Appl. Environ. Microbiol..

[16] Kuratashvili A.Y., Plekhanov N.A., Karpunina L.V., Zadnova S.P. (2024). Systems of Phage Resistance in *Vibrio* Cholerae Strains. Probl. Part. Danger. Infect..

[17] Witte S., Zinsli L.V., Gonzalez-Serrano R., Matter C.I., Loessner M.J., van Mierlo J.T., Dunne M. (2021). Structural and Functional Characterization of the Receptor Binding Proteins of Escherichia Coli O157 Phages EP75 and EP335. Comput. Struct. Biotechnol. J..

[18] Bernard C., Labreuche Y., Diarra C., Daszkowski P., Cahier K., Goudenège D., Lamarche M.G., Whitfield G.B., Lang M., Valencia J. (2025). Adaptive Genomic Plasticity in Large-Genome, Broad-Host–Range *Vibrio* Phages. ISME J..

[19] Holtappels D., Alfenas-Zerbini P., Koskella B. (2023). Drivers and Consequences of Bacteriophage Host Range. FEMS Microbiol. Rev..

[20] Osset-Trenor P., Proft M., Pascual-Ahuir A. (2026). From Gatekeepers to Mitochondrial Mischief: How Bacterial Outer Membrane Proteins Crash the Host Cell Party. FEMS Microbiol. Rev..

[21] Xihui Z., Junshan H., Jiayin X., Luqi T., Pei L., Wei J., Wei Z. (2025). Infection and Resistance Mechanisms of Phage VB_VpM-PP270 in *Vibrio* Parahaemolyticus via Capsular Polysaccharide. iScience.

[22] Li X., Li S., Zhang C., Zhang C., Xu X., Zhou X., Zhao Z. (2024). Autographiviridae Phage HH109 Uses Capsular Polysaccharide for Infection of *Vibrio* Alginolyticus. iScience.

[23] Hu M., Zhang H., Gu D., Ma Y., Zhou X. (2020). Identification of a Novel Bacterial Receptor That Binds Tail Tubular Proteins and Mediates Phage Infection of *Vibrio* Parahaemolyticus. Emerg. Microbes Infect..

[24] Inoue T., Matsuzaki S., Tanaka S. (1995). A 26-KDa Outer Membrane Protein, OmpK, Common to *Vibrio* Species Is the Receptor for a Broad-Host-Range *Vibrio*phage, KVP40. FEMS Microbiol. Lett..

[25] Fan F., Li X., Pang B., Zhang C., Li Z., Zhang L., Li J., Zhang J., Yan M., Liang W. (2017). The Outer-Membrane Protein TolC of *Vibrio* Cholerae Serves as a Second Cell-Surface Receptor for the VP3 Phage. J. Biol. Chem..

[26] Fan F., Li Z., Wang J., Diao B., Liang W., Kan B. (2021). A PolyQ Membrane Protein of *Vibrio* Cholerae Acts as the Receptor for Phage Infection. J. Virol..

[27] Lee H.S., Choi S., Shin H., Lee J.H., Choia S.H. (2014). *Vibrio* Vulnificus Bacteriophage SSP002 as a Possible Biocontrol Agent. Appl. Environ. Microbiol..

[28] Li J., Tian F., Hu Y., Lin W., Liu Y., Zhao F., Ren H., Pan Q., Shi T., Tong Y. (2021). Characterization and Genomic Analysis of BUCT549, a Novel Bacteriophage Infecting *Vibrio* Alginolyticus With Flagella as Receptor. Front. Microbiol..

[29] Wang N., Li C., Zhao J., Yue Y., Shi T., Wang Z., Liang Y., Zhang Y., Wang M. (2025). Enhanced Control of Pathogenic *Vibrio* Spp. in Aquaculture Using Phages Capable of Disrupting Biofilms Outside Their Host Range. Appl. Environ. Microbiol..

[30] Ouyang R., Ongenae V., Muok A., Claessen D., Briegel A. (2024). Phage Fibers and Spikes: A Nanoscale Swiss Army Knife for Host Infection. Curr. Opin. Microbiol..

[31] Tan D., Svenningsen S.L., Middelboe M. (2015). Quorum Sensing Determines the Choice of Antiphage Defense Strategy in *Vibrio* Anguillarum. mBio.

[32] Wang W., Liu J., Guo S., Liu L., Yuan Q., Guo L., Pan S. (2021). Identification of *Vibrio* Parahaemolyticus and *Vibrio* Spp. Specific Outer Membrane Proteins by Reverse Vaccinology and Surface Proteome. Front. Microbiol..

[33] Inoue T., Matsuzaki S., Tanaka S. (1995). Cloning and Sequence Analysis of *Vibrio* Parahaemolyticus OmpK Gene Encoding a 26-KDa Outer Membrane Protein, OmpK, That Serves as Receptor for a Broad-Host-Range *Vibrio*phage, KVP40. FEMS Microbiol. Lett..

[34] Xu D., Zhang J., Liu J., Xu J., Zhou H., Zhang L., Zhu J., Kan B. (2014). Outer Membrane Protein OmpW Is the Receptor for Typing Phage VP5 in the *Vibrio* Cholerae O1 El Tor Biotype. J. Virol..

[35] Ningqiu L., Junjie B., Shuqin W., Xiaozhe F., Haihua L., Xing Y., Cunbin S. (2008). An Outer Membrane Protein, OmpK, Is an Effective Vaccine Candidate for *Vibrio* Harveyi in Orange-Spotted Grouper (*Epinephelus coioides*). Fish. Shellfish Immunol..

[36] Sperandio V., Bailey C., Girón J.A., DiRita V.J., Silveira W.D., Vettore A.L., Kaper J.B. (1996). Cloning and Characterization of the Gene Encoding the OmpU Outer Membrane Protein of *Vibrio* Cholerae. IJSIM.

[37] Nakasone N., Iwanaga M., Nakasone N., Iwanaga M. (1998). Characterization of Outer Membrane Protein OmpU of *Vibrio* Cholerae O1. Infect. Immun..

[38] Zhao X., Liu Y., Yan F., Lin Z., Zhao Y., Chen X., Zhang Y. (2024). OmpU and OmpC Are the Key OMPs for Litopenaeus Vannamei Hemocyanin Recognizes *Vibrio* Parahaemolyticus. Fish. Shellfish Immunol..

[39] Gulati A., Kumar R., Mukhopadhaya A. (2019). Differential Recognition of *Vibrio* Parahaemolyticus OmpU by Toll-like Receptors in Monocytes and Macrophages for the Induction of Proinflammatory Responses. Infect. Immun..

[40] Wang Q., Chen J., Liu R., Jia J. (2011). Identification and Evaluation of an Outer Membrane Protein OmpU from a Pathogenic *Vibrio* Harveyi Isolate as Vaccine Candidate in Turbot (*Scophthalmus maximus*). Lett. Appl. Microbiol..

[41] Sharma A., Yadav S.P., Sarma D., Mukhopadhaya A. (2022). Modulation of Host Cellular Responses by Gram-Negative Bacterial Porins. Adv. Protein Chem. Struct. Biol..

[42] Xuan G., Lu D., Lin H., Wang Y., Wang J. (2024). Outer Membrane Vesicle Production by Escherichia Coli Enhances Its Defense against Phage Infection. Microorganisms.

[43] Karthikeyan A., Javaid A., Tabassum N., Kim T.H., Kim Y.M., Jung W.K., Khan F. (2006). Bacterial Extracellular Membrane Vesicles as Multifunctional Defense Systems: Roles in Immune Evasion, Phage Interactions, and Antimicrobial Resistance. Mol. Biol. Rep..

[44] Bołoz A., Lannoy V., Olszak T., Drulis-Kawa Z., Augustyniak D. (2025). Interplay Between Bacterial Extracellular Vesicles and Phages: Receptors, Mechanisms, and Implications. Viruses.

[45] Potapova A., Garvey W., Dahl P., Guo S., Chang Y., Schwechheimer C., Trebino M.A., Floyd K.A., Phinney B.S., Liu J. (2024). Outer Membrane Vesicles and the Outer Membrane Protein OmpU Govern *Vibrio* Cholerae Biofilm Matrix Assembly. mBio.

[46] Echeverría-Bugueño M., Hernández M., Avendaño-Herrera R. (2024). Proteomic Analysis of the Fish Pathogen *Vibrio* Ordalii Strain Vo-LM-18 and Its Outer Membrane Vesicles. Animals.

[47] Li J., Guo A., Huang S., Azam F., Sun X., Zhang J., Long L., Zhang S. (2024). Outer Membrane Vesicles Produced by Coral-Associated *Vibrio* Coralliilyticus Inhibit Bacteriophage Infection and Its Ecological Implications. Microbiol. Res..

[48] Reyes-Robles T., Dillard R.S., Cairns L.S., Silva-Valenzuela C.A., Housman M., Ali A., Wright E.R., Camilli A. (2018). *Vibrio* Cholerae Outer Membrane Vesicles Inhibit Bacteriophage Infection. J. Bacteriol..

[49] Saeju P., Naknaen A., Sukonthamarn P., Tassanakajon A., Nonejuie P., Chaikeeratisak V. (2025). Enhanced Efficiency of Virulent and Temperate Phage Combination Mediated through Bacterial Membrane Vesicles. J. Virol..

[50] Zhou G., Wang Q., Wang Y., Wen X., Peng H., Peng R., Shi Q., Xie X., Li L. (2023). Outer Membrane Porins Contribute to Antimicrobial Resistance in Gram-Negative Bacteria. Microorganisms.

[51] Benz R., Benz R. (2021). Prokaryotic and Eukaryotic Porins: Comparison of Structure and Function. Developmental Biology in Prokaryotes and Lower Eukaryotes.

[52] Li L.-H., Wu C.-M., Chang C.-L., Huang H.-H., Wu C.-J., Yang T.-C. (2022). ΣP-NagA-L1/L2 Regulatory Circuit Involved in ΔompA299-356-Mediated Increase in β-Lactam Susceptibility in Stenotrophomonas Maltophilia. Microbiol. Spectr..

[53] Leprince A., Mahillon J. (2023). Phage Adsorption to Gram-Positive Bacteria. Viruses.

[54] Che J., Fang Q., Hu S., Liu B., Wang L., Fang X., Li L., Luo T., Bao B. (2024). The Impact of Vp-Porin, an Outer Membrane Protein, on the Biological Characteristics and Virulence of *Vibrio* Parahaemolyticus. Biology.

[55] Chamblee J.S., Ramsey J., Chen Y., Maddox L.T., Ross C., To K.H., Cahill J.L., Young R. (2022). Endolysin Regulation in Phage Mu Lysis. mBio.

[56] Gigante A.M., Olivença F., Catalão M.J., Leandro P., Moniz-Pereira J., Filipe S.R., Pimentel M. (2021). The Mycobacteriophage Ms6 LysB N-Terminus Displays Peptidoglycan Binding Affinity. Viruses.

[57] Nair G., Jain V. (2024). An Intramolecular Cross-Talk in D29 Mycobacteriophage Endolysin Governs the Lytic Cycle and Phage-Host Population Dynamics. Sci. Adv..

[58] Peng Y., Pang H., Zheng J., Zhou J., Chen W., Yang F., Xiao H., Liu H. (2026). Structure of a Brochothrix Thermosphacta Bacteriophage Reveals Cell Wall Adsorption Mechanism in Gram-Positive Infecting Siphophages. Nat. Commun..

[59] Dicks L.M.T., Vermeulen W. (2024). Bacteriophage–Host Interactions and the Therapeutic Potential of Bacteriophages. Viruses.

[60] Ebrahimi A., Ergün T., Kaygusuz İzgördü Ö., Darcan C., Avci H., Öztürk B., Güner H.R., Ghorbanpoor H., Doğan Güzel F. (2023). Revealing the Single-Channel Characteristics of OprD (OccAB1) Porins from Hospital Strains of Acinetobacter Baumannii. Eur. Biophys. J..

[61] Noori F.A., Mushtaq M.S., Latif M.S., Hamdani S.D.A., Babar M.M. (2024). Bacteriophage Therapy in GIT Infections—A Clinical Review. NUST J. Nat. Sci..

[62] Mascolo E., Adhikari S., Caruso S.M., DeCarvalho T., Folch Salvador A., Serra-Sagristà J., Young R., Erill I., Curtis P.D. (2022). The Transcriptional Regulator CtrA Controls Gene Expression in Alphaproteobacteria Phages: Evidence for a Lytic Deferment Pathway. Front. Microbiol..

[63] Gambino M., Sørensen M.C.H. (2024). Flagellotropic Phages: Common yet Diverse Host Interaction Strategies. Curr. Opin. Microbiol..

[64] Lin H.-N., Huang X.-H., Miao X.-J., Hu W.-L., Lou Y.-L., Xie D.-L., van der Veen S.E. (2024). Quorum Sensing: An Emerging Role for *Vibrio* Infection and Host Defense. Infect. Microbes Dis..

[65] Chang J., Zhou Y., Zhang M., Li X., Zhang N., Luo X., Ni B., Wu H., Lu R., Zhang Y. (2024). CalR Inhibits the Swimming Motility and Polar Flagellar Gene Expression in *Vibrio* Parahaemolyticus. J. Microbiol..

[66] Lohrmann C., Holm C., Datta S.S. (2024). Influence of Bacterial Swimming and Hydrodynamics on Attachment of Phages. Soft Matter.

[67] Ostenfeld L.J., Sørensen A.N., Neve H., Vitt A., Klumpp J., Sørensen M.C.H. (2024). A Hybrid Receptor Binding Protein Enables Phage F341 Infection of Campylobacter by Binding to Flagella and Lipooligosaccharides. Front. Microbiol..

[68] Bali K., McCoy R., Lu Z., Treiber J., Savva A., Kaminski C.F., Salmond G., Salleo A., Mela I., Monson R. (2023). Multiparametric Sensing of Outer Membrane Vesicle-Derived Supported Lipid Bilayers Demonstrates the Specificity of Bacteriophage Interactions. ACS Biomater. Sci. Eng..

[69] Fernandez-Martinez D., Kong Y., Goussard S., Zavala A., Gastineau P., Rey M., Ayme G., Chamot-Rooke J., Lafaye P., Vos M. (2024). Cryo-EM Structures of Type IV Pili Complexed with Nanobodies Reveal Immune Escape Mechanisms. Nat. Commun..

[70] Tang Y., Li J., Wang Y., Song Z., Ying H., Kong L., Jiao X., Huang J. (2022). Campylobacter Jejuni Developed the Resistance to Bacteriophage CP39 by Phase Variable Expression of 06875 Encoding the CGPTase. Viruses.

[71] Zeng X., Liang S., Dong J., Gao G., Hu Y., Sun Y. (2024). The Trade-off of *Vibrio* Parahaemolyticus between Bacteriophage Resistance and Growth Competitiveness. Front. Microbiol..

[72] Ts A.S., Sanil N. (2024). Whole Genome Sequencing of *Vibrio* Alginolyticus: Insights into Virulence and Antimicrobial Resistance: Whole Genome Sequencing of Vibrio Alginolyticus. Fish. Technol..

[73] Skliros D., Kalatzis P.G., Kalloniati C., Komaitis F., Papathanasiou S., Kouri E.D., Udvardi M.K., Kokkari C., Katharios P., Flemetakis E. (2021). The Development of Bacteriophage Resistance in *Vibrio* Alginolyticus Depends on a Complex Metabolic Adaptation Strategy. Viruses.

[74] Lv T., Dai F., Zhuang Q., Zhao X., Shao Y., Guo M., Lv Z., Li C., Zhang W. (2020). Outer Membrane Protein OmpU Is Related to Iron Balance in *Vibrio* Alginolyticus. Microbiol. Res..

[75] Fineran P.C. (2019). Resistance Is Not Futile: Bacterial ‘Innate’ and CRISPR-Cas ‘Adaptive’ Immune Systems. Microbiology.

[76] Picton D.M., Luyten Y.A., Morgan R.D., Nelson A., Smith D.L., Dryden D.T.F., Hinton J.C.D., Blower T.R. (2021). The Phage Defence Island of a Multidrug Resistant Plasmid Uses Both BREX and Type IV Restriction for Complementary Protection from Viruses. Nucleic Acids Res..

[77] Ambroa A., Blasco L., López M., Pacios O., Bleriot I., Fernández-García L., González de Aledo M., Ortiz-Cartagena C., Millard A., Tomás M. (2022). Genomic Analysis of Molecular Bacterial Mechanisms of Resistance to Phage Infection. Front. Microbiol..

[78] Watson B.N.J., Vercoe R.B., Salmond G.P.C., Westra E.R., Staals R.H.J., Fineran P.C. (2019). Type I-F CRISPR-Cas Resistance against Virulent Phage Infection Triggers Abortive Infection and Provides Population-Level Immunity. bioRxiv.

[79] Yikun P. (2025). The CRISPR-Cas, PAgo, and CBASS Mediated Abortive Infection and Its Potential Application. Theor. Nat. Sci..

[80] Mayo-Muñoz D., Smith L.M., Garcia-Doval C., Malone L.M., Harding K.R., Jackson S.A., Hampton H.G., Fagerlund R.D., Gumy L.F., Fineran P.C. (2022). Type III CRISPR-Cas Provides Resistance against Nucleus-Forming Jumbo Phages via Abortive Infection. Mol. Cell.

[81] Kim H.J., Kwon J., Lee C.H., Kim J.H., Park S.C., Kim S.G. (2025). Comparison of the Genomes of *Vibrio* Phages PVco-5 and PVco-7: Implications for Phage-Host Interactions. Arch. Virol..

[82] Zhang L., Wang H., Zeng J., Cao X., Gao Z., Liu Z., Li F., Wang J., Zhang Y., Yang M. (2024). Cas1 Mediates the Interference Stage in a Phage-Encoded CRISPR–Cas System. Nat. Chem. Biol..

[83] Maxwell K.L. (2019). Phages Tune in to Host Cell Quorum Sensing. Cell.

[84] Lopatina A., Tal N., Sorek R. (2020). Abortive Infection: Bacterial Suicide as an Antiviral Immune Strategy. Annu. Rev. Virol..

[85] Seed K.D., Faruque S.M., Mekalanos J.J., Calderwood S.B., Qadri F., Camilli A. (2012). Phase Variable O Antigen Biosynthetic Genes Control Expression of the Major Protective Antigen and Bacteriophage Receptor in *Vibrio* Cholerae O1. PLoS Pathog..

[86] Zhang T., Ji S., Zhang M., Wu F., Li X., Luo X., Huang Q., Li M., Zhang Y., Lu R. (2024). Effect of Capsular Polysaccharide Phase Variation on Biofilm Formation, Motility and Gene Expression in Vibrio Vulnificus. Gut Pathog..

[87] Lin L.-C., Tsai Y.-C. (2022). Isolation and Characterization of a *Vibrio* Owensii Phage Phi50-12. Sci. Rep..

[88] Netter Z., Dunham D.T., Seed K.D. (2023). Adaptation to Bile and Anaerobicity Limits *Vibrio* Cholerae Phage Adsorption. mBio.

[89] Li W., Zhu R., Shi K., Ding H., Ren S., Xu S., Zhao J., Jiao N., Yang Y., Zhang R. (2026). Distinct Ecophysiological Characteristics of Two Genetically Similar *Vibrio*phages. Mar. Life Sci. Technol..

[90] Zhang C., Li X., Li S., Yin H., Zhao Z. (2024). Characterization and Genomic Analysis of a Broad-Spectrum Lytic Phage PG288: A Potential Natural Therapy Candidate for *Vibrio* Infections. Virus Res..

[91] Kang J., Chang Y. (2023). Characterization of a *Vibrio* Parahaemolyticus-Targeting Lytic Bacteriophage SSJ01 and Its Application in Artificial Seawater. Food Sci. Biotechnol..

[92] Piel D., Bruto M., Labreuche Y., Blanquart F., Goudenège D., Barcia-Cruz R., Chenivesse S., Panse S.L., James A., Dubert J. (2022). Phage-Host Coevolution in Natural Populations. Nat. Microbiol..

[93] Misol G.N., Kokkari C., Katharios P. (2020). Biological and Genomic Characterization of a Novel Jumbo Bacteriophage, VB_VhaM_pir03 with Broad Host Lytic Activity against *Vibrio* Harveyi. Pathogens.

[94] Chen D., Wang Z., Li X., Du H., Zhang K., Cao S., Lu J., Zhao S., Wang H., Li Y. (2024). Biological Properties of *Vibrio* Parahaemolyticus Lytic Phages and Transcriptome Analysis of Their Interactions with the Host. Aquac. Rep..

[95] Kannoly S., Singh K., Juman N., Islam Z.M., Quiroga I.C., Singh A., Dennehy J.J., Kannoly S., Singh K., Juman N. (2025). Translation Efficiency Impacts Phage Lysis Timing and Its Precision in Single Cells. bioRxiv.

[96] Dominguez-Mirazo M., Harris J.D., Demory D., Weitz J.S. (2024). Accounting for Cellular-Level Variation in Lysis: Implications for Virus-Host Dynamics. bioRxiv.

[97] Ahammad T., Khan R.H., Sahu I.D., Drew D.L., Faul E., Li T., McCarrick R.M., Lorigan G.A. (2021). Pinholin S21 Mutations Induce Structural Topology and Conformational Changes. Biochim. Biophys. Acta Biomembr..

[98] Zhang R., He J., Yao F., Chen F., Wei H., Huang S., Li Y. (2026). Discovery of Phage Lysin against *Vibrio* Parahaemolyticus and Characterization of Its Antibacterial Activity. Int. J. Food Microbiol..

[99] Huang D., Xia R., Chen C., Liao J., Chen L., Wang D., Alvarez P.J.J., Yu P. (2024). Adaptive Strategies and Ecological Roles of Phages in Habitats under Physicochemical Stress. Trends Microbiol..

[100] Sae-Ueng U., Bhunchoth A., Phironrit N., Treetong A., Sapcharoenkun C., Chatchawankanphanich O., Leartsakulpanich U., Chitnumsub P. (2022). Thermoresponsive C22 Phage Stiffness Modulates the Phage Infectivity. Sci. Rep..

[101] Cahier K., Piel D., Barcia-Cruz R., Goudenège D., Wegner K.M., Monot M., Romalde J.L., Le Roux F. (2023). Environmental *Vibrio* Phage-Bacteria Interaction Networks Reflect the Genetic Structure of Host Populations. Environ. Microbiol..

[102] Rizvi S.M.D., Lila A.S.A., Moin A., Syed S., Khafagy E.S., Askoura M., Rajab A.A.H., Hegazy W.A.H. (2025). Bacteriophage Resurrection: Innovative Impacts in Medicine, Biotechnology, and Environmental Solutions. Sci. Afr..

[103] Breitbart M., Bonnain C., Malki K., Sawaya N.A. (2018). Phage Puppet Masters of the Marine Microbial Realm. Nat. Microbiol..

[104] Zhao A., Sun J., Liu Y. (2023). Understanding Bacterial Biofilms: From Definition to Treatment Strategies. Front. Cell. Infect. Microbiol..

[105] Castillo D., Rørbo N., Jørgensen J., Lange J., Tan D., Kalatzis P.G., Svenningsen S.L., Middelboe M. (2019). Phage Defense Mechanisms and Their Genomic and Phenotypic Implications in the Fish Pathogen *Vibrio* Anguillarum. FEMS Microbiol. Ecol..

[106] Kauffman K.M., Chang W.K., Brown J.M., Hussain F.A., Yang J., Polz M.F., Kelly L. (2022). Resolving the Structure of Phage–Bacteria Interactions in the Context of Natural Diversity. Nat. Commun..

[107] Trajkovic K., Valdez C., Ysselstein D., Krainc D. (2019). Fluctuations in Cell Density Alter Protein Markers of Multiple Cellular Compartments, Confounding Experimental Outcomes. PLoS ONE.

[108] Borodovich T., Shkoporov A.N., Ross R.P., Hill C. (2022). Phage-Mediated Horizontal Gene Transfer and Its Implications for the Human Gut Microbiome. Gastroenterol. Rep..

[109] Wang X., Mahmoud A.A., Elafify M., Zhang S., Liao X., Ding T., Ahn J. (2026). Regulatory Challenges and Industrial Applications of Phages and Phage-Encoded Enzymes for Food Safety. Food Control.

[110] Dwivedi Y.K., Jeyaraj A., Hughes L., Davies G.H., Ahuja M., Albashrawi M.A., Al-Busaidi A.S., Al-Sharhan S., Al-Sulaiti K.I., Altinay L. (2024). “Real Impact”: Challenges and Opportunities in Bridging the Gap between Research and Practice—Making a Difference in Industry, Policy, and Society. Int. J. Inf. Manag..

[111] Nichols A.L., Edlund J.E. (2015). Practicing What We Preach (and Sometimes Study): Methodological Issues in Experimental Laboratory Research. Rev. Gen. Psychol..

[112] Cui L., Kiga K., Kondabagil K., Węgrzyn A. (2024). Current and Future Directions in Bacteriophage Research for Developing Therapeutic Innovations. Sci. Rep..

[113] Shao H., Deng Y., Shi Y., Duan Y. (2026). Engineered Bacteriophages: A Next-Generation Platform for Precision Antimicrobials and Therapeutics. Viruses.

[114] Lim K.C., Yusoff F.M., Natrah F.M.I., De Zoysa M., Md Yasin I.S., Yaminudin J., Karim M. (2025). Biological Strategies in Aquaculture Disease Management: Towards a Sustainable Blue Revolution. Aquac. Fish..

[115] Steensen K., Séneca J., Bartlau N., Yu X.A., Hussain F.A., Polz M.F. (2024). Tailless and Filamentous Prophages Are Predominant in Marine Vibrio. ISME J..

[116] González-Gómez J.P., Soto-Rodriguez S.A., Gomez-Gil B., Serrano-Hernández J.M., Lozano-Olvera R., López-Cuevas O., Castro-del Campo N., Chaidez C. (2023). Effect of Phage Therapy on Survival, Histopathology, and Water Microbiota of Penaeus Vannamei Challenged with *Vibrio* Parahaemolyticus Causing Acute Hepatopancreatic Necrosis Disease (AHPND). Aquaculture.

[117] Yang Y., Dufault-Thompson K., Yan W., Cai T., Xie L., Jiang X. (2024). Large-Scale Genomic Survey with Deep Learning-Based Method Reveals Strain-Level Phage Specificity Determinants. Gigascience.

[118] Doud M.B., Robertson J.M., Strathdee S.A. (2025). Optimizing Phage Therapy with Artificial Intelligence: A Perspective. Front. Cell. Infect. Microbiol..

